# Overlapping Functions of Argonaute Proteins in Patterning and Morphogenesis of *Drosophila* Embryos

**DOI:** 10.1371/journal.pgen.0020134

**Published:** 2006-08-25

**Authors:** Wibke J Meyer, Silke Schreiber, Yi Guo, Thorsten Volkmann, Michael A Welte, H. Arno J Müller

**Affiliations:** 1 Institut für Genetik, Heinrich-Heine Universität, Düsseldorf, Germany; 2 Department of Biology, Brandeis University, Waltham, Massachusetts, United States of America; The Jackson Laboratory, United States of America

## Abstract

Argonaute proteins are essential components of the molecular machinery that drives RNA silencing. In *Drosophila,* different members of the Argonaute family of proteins have been assigned to distinct RNA silencing pathways. While Ago1 is required for microRNA function, Ago2 is a crucial component of the RNA-induced silencing complex in siRNA-triggered RNA interference. *Drosophila* Ago2 contains an unusual amino-terminus with two types of imperfect glutamine-rich repeats (GRRs) of unknown function. Here we show that the GRRs of Ago2 are essential for the normal function of the protein. Alleles with reduced numbers of GRRs cause specific disruptions in two morphogenetic processes associated with the midblastula transition: membrane growth and microtubule-based organelle transport. These defects do not appear to result from disruption of siRNA-dependent processes but rather suggest an interference of the mutant Ago2 proteins in an Ago1-dependent pathway. Using loss-of-function alleles, we further demonstrate that Ago1 and Ago2 act in a partially redundant manner to control the expression of the segment-polarity gene *wingless* in the early embryo. Our findings argue against a strict separation of Ago1 and Ago2 functions and suggest that these proteins act in concert to control key steps of the midblastula transition and of segmental patterning.

## Synopsis

Cells employ diverse mechanisms to control the activity of their genes, and over the last ten years, a new strategy for gene regulation called RNA silencing has been discovered. Central components responsible for RNA silencing are Argonaute proteins. While many of the molecular properties of Argonaute proteins have been uncovered, little is known about their function in living organisms. In *Drosophila,* it has been puzzling that mutations in individual Argonaute proteins lead to surprisingly mild defects in development, although RNA silencing had been suggested to play major roles in gene regulation. Meyer and coworkers describe that Ago1 and Ago2, two Argonaute family members in *Drosophila,* function in a redundant fashion. Previously, these two proteins were demonstrated to mediate distinct pathways of RNA silencing. The authors show that in early embryos Ago1 and Ago2 work together in two fundamental processes: the generation of polarity within cells, by controlling the unequal distributions of proteins and cell organelles, and the polarity of tissues, by modulating an important cell-cell signaling pathway. These results connect the activity of Argonaute proteins and by extension the mechanisms of RNA silencing with central problems of cell and developmental biology, namely the regulation of polarity in cells and tissues.

## Introduction

RNA silencing represents an important regulatory mechanism of gene expression, in which short RNAs regulate mRNA stability and translation or control transcription via chromatin modification [1]. Such short RNAs include small interfering RNAs (siRNAs) and microRNAs (miRNA). Small RNAs are produced from larger precursors by RNase III–type endonucleases, called Dicer [2,3]. Processed small RNAs are incorporated into the RNA-induced silencing complex (RISC, also called miRNP in the case of miRNAs) [4,5]. Active RISC either catalyzes the cleavage of the cognate target mRNA or interferes with its translation. RNA silencing directed by siRNAs has been implicated in heterochromatic silencing in various organisms from yeast to humans [6]. miRNAs are involved in a variety of biological processes such as cell proliferation, cell death, developmental timing, and embryonic patterning [7].

Biochemical and genetic analyses have revealed that the molecular mechanisms underlying RNA silencing crucially require proteins from the Argonaute family [8]. Argonaute proteins are essential components of RISC and represent the catalytic activity of RISC in both the miRNA and the siRNA pathway [9–14]. Argonaute proteins are highly conserved and share at least two functional domains, a PAZ and a PIWI domain [8]. The PAZ domain forms a nucleic acid–binding pocket and binds small RNAs [15,16]. The PIWI domain shares structural similarities with ribonucleases and contains an activity that degrades cognate RNAs [13,14,17–19].

The *Drosophila* genome encodes five Argonaute family members: Aubergine (Aub), Piwi, Ago1, Ago2, and Ago3 [8,20,21]. Ago2 differs from the other *Drosophila* Argonautes in that it has a unique amino-terminal extension rich in glutamines (37% of all residues are glutamine). Almost two-thirds of this domain is made up of tandem copies of two types of glutamine-rich repeats (GRRs). In other proteins, glutamine-rich domains can promote protein aggregation such as the PolyQ domains in the mutant forms of Huntingtin linked to neurodegeneration or the PrD domains of many yeast prions [22,23]. For Ago2, however, the functional significance of this domain is unknown.

In *Drosophila,* siRNA-induced and miRNA-induced silencing vary in their requirement for different Argonaute and Dicer genes. Ago1 is required for miRNA-induced RNA silencing but is dispensable for siRNA-triggered RNA cleavage in vitro [11]. A combination of biochemical and genetic approaches has demonstrated an essential function of Ago2 for RNAi [9,11], but depletion of Ago2 does not impair miRNA-directed RNA cleavage in vitro. A similar distinction has been detected at the level of Dicer: *Dicer-1 (Dcr-1)* mutants are defective in processing pre-miRNAs, while *Dicer-2 (Dcr-2)* mutants are defective in processing siRNA precursors [24]. The fact that null mutations in *Dcr-2* are homozygous viable and fertile suggests that siRNA-triggered RNAi is not essential for normal development. In contrast, homozygous *Dcr-1* null mutants are lethal supporting the model that regulation through miRNAs is a crucial mechanism during embryogenesis.

We have characterized the maternal-effect mutation *drop out (dop),* which causes specific developmental defects at the midblastula transition. The mutant embryos show a transient block in membrane growth and fail to undergo a developmental switch in the microtubule-based polarized transport of lipid droplets. Surprisingly, we find that *dop* mutations represent special alleles of *ago2*. Two independently generated *dop* alleles reduce the copy number of the GRRs, providing the first evidence of a functional role of this domain. These mutations render Ago2 only partially defective in siRNA responses. However, these alleles interact genetically with Ago1, suggesting the possibility of crosstalk between Ago1- and Ago2-mediated pathways. This conclusion is further supported by double-mutant analysis using loss-of-function alleles of *ago2* and *ago1;* we demonstrate that the two gene products function redundantly in embryonic patterning. Our results reveal novel functions of Argonaute proteins in early embryogenesis and suggest a regulatory role for the GRR domain of Ago2.

## Results

### The *dop* Mutation Affects Membrane Growth during Cellularization

To dissect the mechanisms that establish epithelial cell polarity, we reexamined the previously described female-sterile mutation *dop* [25]. Flies homozygous or hemizygous for *dop^1^* are viable, but embryos from *dop^1^* homozygous mothers do not hatch and display defects in larval cuticle formation, reminiscent of *bazooka* or *crumbs* alleles, mutations that disturb the formation of the embryonic epidermis (see below). Using videomicroscopy of living embryos, we found that these embryos display severe morphological defects about 3 h after fertilization, at the time of the midblastula transition (MBT).

**Figure 1:**
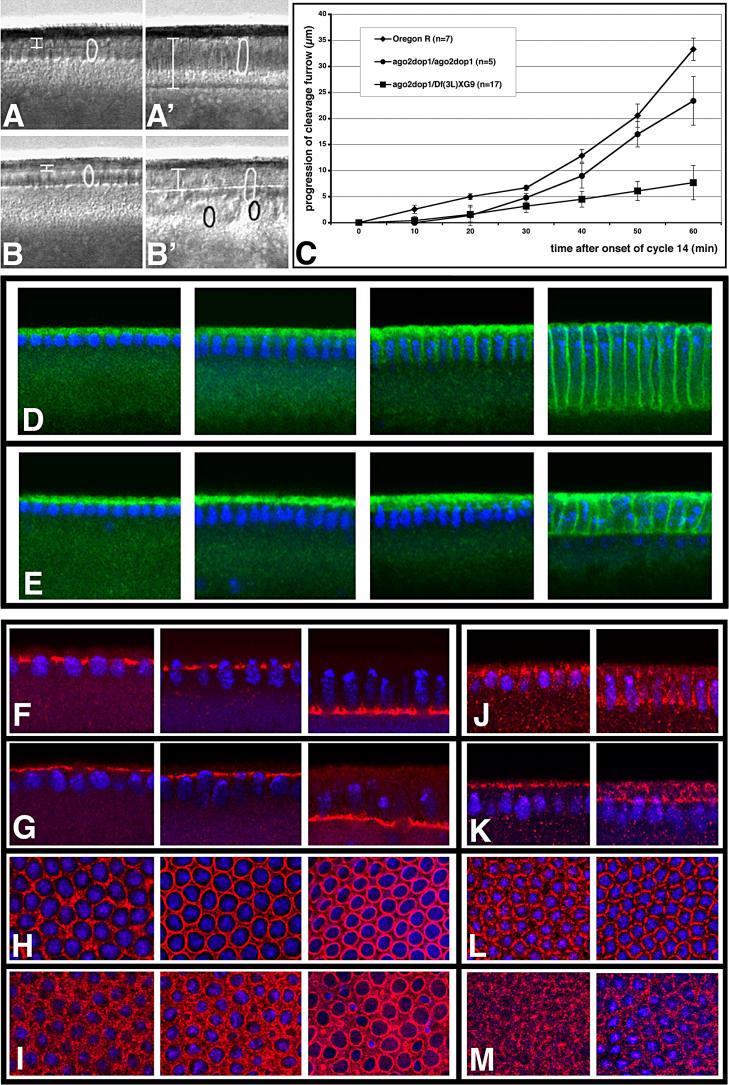
*dop^1^* Affects Cell Formation in the Early Embryo (A–C) Membrane extension is strongly reduced in *dop^1^* embryos. (A, A′) Frames taken from a video-sequence of a wild-type embryo. In the wild-type, membranes grow slowly for the first 30 min of cycle 14 interphase [slow phase (A)], and then growth speeds up considerably [fast phase (A′)]. (B, B′) *dop^1^* mutant embryo from a video-sequence at corresponding time points. The shape of the nuclei (circles) and the extent of furrow progression are indicated (bars). (C) The progression of the cleavage furrow was plotted against the time after the beginning of mitotic cycle 14. Note that compared to the wild-type furrow progression is significantly slower in embryos derived from homozygous (*dop^1^/dop^1^*) or hemizygous *[dop^1^/Df(3L)XG9] dop^1^* females, within the first 30 min of cellularization. In *dop^1^* homozygotes, membranes advanced 0.07 μm/min during slow phase, compared to 0.25 μm/min in the wild-type. In hemizygous *dop^1^* embryos, membrane formation was even more severely impaired; it was slower during both slow and fast phase. (D, E) Immunostaining of the endogenous membrane protein Neurotactin (Nrt; green) and DNA (blue) in wild-type (D) and *dop^1^* (E) mutant embryos during progression of cell formation (from left to right panel). While Nrt associates with the egg cortex in both cases, Nrt positive cleavage membranes in *dop^1^* mutant embryos are absent until fast phase. (F–I) Immunostaining with Slam antibodies (red) indicates the presence of furrow canals in wild-type (F, H) and *dop^1^* (G, H) mutant embryos (F, G: optical cross section; H, I: tangential optical section) at progressively older stages of cell formation. (F, H) In the wild-type, formation of the furrow canal can be seen as a regular array of loop-like structures beginning with nuclear elongation. (G, I) In *dop^1^* mutant embryos, Slam localization does not resolve into this regular array and forms an unevenly distributed network apical to the nuclei. (J–M) Immunostaining with Arm antibodies (red) indicates the positioning of the basal junctions; two timepoints are shown in each panel: beginning of slow phase to the left and beginning of fast phase to the right. (J, L) In the wild-type, Arm accumulates adjacent to the furrow canals and forms a honeycomb pattern as seen in surface view. (K, M) In *dop^1^* mutant embryos, Arm remains apical and does not accumulate in basal junctions. Fixed embryos were staged by the extent of nuclear elongation, as described by Lecuit et al. [30].

The MBT is marked by the onset of substantial zygotic transcription and is accompanied by dramatic morphological changes. One of the most striking MBT-specific processes in *Drosophila* is cell formation, and it is this process that is abnormal in *dop* mutants. In the wild-type, the first 13 cleavage divisions of the zygote take place without cytokinesis, and during cycle 14, the time of the MBT, polarized growth of the plasma membrane transforms the syncytial embryo into the polarized blastoderm epithelium [26]. In the *dop^1^* mutant embryos, we detected no significant differences to the wild-type up to cycle 13 (not shown). But the initiation of membrane growth in cycle 14 was significantly delayed (Figure 1A–1C). The defect in membrane growth was also evident by immunolabeling for an endogenous marker for cleavage membranes, Neurotactin (Nrt) (Figure 1D and 1E). *dop^1^* represents the first example of a maternal-effect mutation that specifically affects morphogenetic events at the MBT.

Cell formation is initiated by the generation of two membrane subdomains: the furrow canal and the basal junction [27]. In *dop^1^* embryos, Slam, a marker for the furrow canal [28], did not accumulate in between the individual nuclei but remained associated with the egg cortex in an irregular pattern throughout the initial stages of cell formation (Figure 1F–1I). Similarly, Arm, a marker for basal junctions, failed to accumulate in a typical honeycomb-like pattern but remained diffusely distributed over the egg cortical cytoplasm (Figure 1J–1M). We conclude that *dop^1^* affects early morphological processes during the MBT, namely, initiation of the furrow canal, formation of basal junctions, and membrane growth.

The morphogenetic events during the MBT in *Drosophila* are controlled by a limited number of zygotic genes [29]. We therefore tested whether the *dop^1^* phenotype results from defective expression of Slam, a known zygotic regulator of cellularization. Zygotic mutants for *slam* display a delay in cellularization very similar to the one of *dop^1^* embryos [28,30]. Yet levels of both *slam* transcript and of Slam protein were normal in *dop^1^* embryos, as were their spatial expression patterns (Figure 1F–1I and unpublished data). Thus, the phenotype of *dop^1^* embryos does not reflect a defect in the regulation of *slam* expression.

Membrane growth during cellularization requires an intact microtubule cytoskeleton [31,32]. Thus, the cellularization defects in *dop^1^* might indicate problems with microtubule-based transport. Since the mechanisms of microtubule transport during cellularization are not well understood, we instead examined a well-defined microtubule-based transport process in the early embryo, the motion of lipid droplets.

### *dop^1^* Embryos Display Aberrant Microtubule-Based Transport of Lipid Droplets

**Figure 2:**
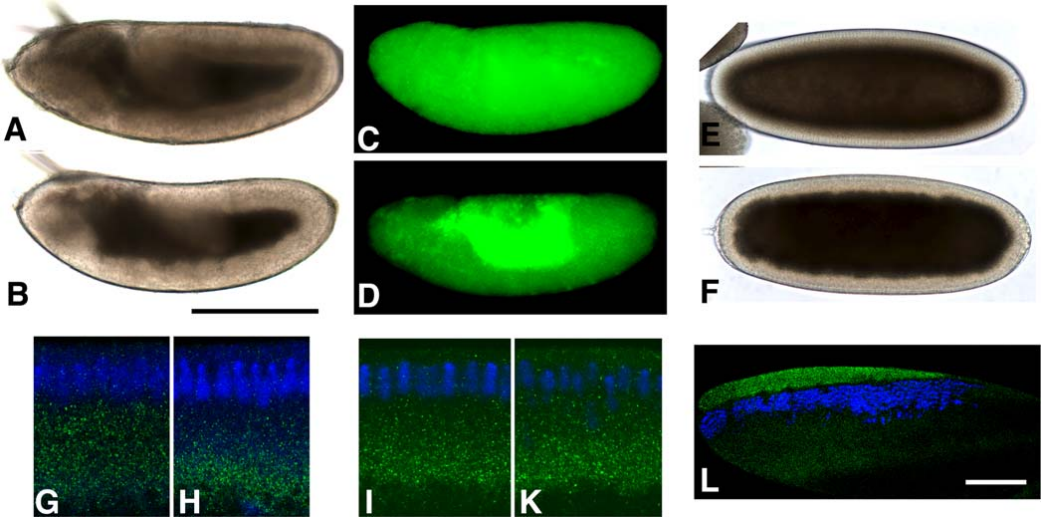
*dop^1^* Compromises Polarized Microtubule-Based Transport (A, B) During germband extension, the periphery of *dop^1^* embryos (B) is much more transparent than that of wild-type embryos (A). (C, D) Lipid droplets (green) were stained with the droplet-specific fluorescent dye Nile Red, and their distribution was recorded by epifluorescence microscopy. In the wild-type, lipid droplets are found throughout the periphery. In the mutant, lipid droplets are accumulated around the central yolk. (E–H) Males homozygously deleted for *halo* were crossed to *dop^1^* heterozygous (E, G) or homozygous (F, H) females to generate embryos with reduced *halo* expression. In these embryos, droplet distribution was assessed by overall transparency (E, F) or staining for the regulator Klar (green) (G, H). In late cycle 14, embryos from *dop^1^* homozygous mothers have a more transparent periphery and tighter basal accumulation of Klar puncta. (I, K) Overall expression and distribution of Klar (green) is very similar in wild-type (I) and *ago2^dop1^* embryos (K). (L) In centrifuged early embryos, lipid droplets accumulate in a distinct layer, just above nuclei (blue). In centrifuged *dop^1^* embryos, Klar (green) is highly enriched in the droplet layer, just as in the wild-type [36], indicating that it is physically associated with the droplets. Scale bars represent 200 μm in (A) and 80 μm in (L). (G–L) Nuclei (blue) were labeled with Hoechst 33258.

When examined by videomicroscopy, *dop^1^* embryos were abnormally transparent from gastrulation onward (Figure 2A and 2B). Such altered transparency can be a signature of mislocalized lipid droplets, as cytoplasm filled with lipid droplets is opaque [33,34]. In the wild-type, lipid droplets are present throughout the embryo periphery after gastrulation. In contrast, in *dop^1^* embryos, they were highly enriched basally, around the yolk sac (Figure 2C and 2D).

In the wild-type, droplet distribution along the apical-basal axis is temporally coordinated with cellularization: droplets are located throughout the periphery in syncytial stages and are transported basally early during cycle 14 and back apically during gastrulation [33]. As a result, the periphery first becomes transparent (cytoplasmic clearing) and then turns opaque again. Because wild-type and *dop^1^* embryos displayed very similar transparency up until the end of cycle 14, the initial basal droplet transport appeared normal. To determine when the difference in droplet transport between these genotypes arises, we employed embryos in which expression of the directionality determinant Halo was reduced. Under these conditions, basal transport is slower and less complete [35]. Embryos that expressed only a single copy of *halo* appeared similar early in cycle 14 whether they were derived from *dop^1^* homozygous or heterozygous mothers (not shown), but late in cycle 14, lipid droplets in embryos from *dop^1^* homozygotes accumulated more basally (Figure 2E–2H), as judged by both higher embryo transparency and tighter basal accumulation of Klar, a droplet-associated regulator [36]. Thus, droplet distribution in *dop^1^* becomes abnormal late in cycle 14, and droplets fail to switch from basal to apical transport.

The abnormal droplet distribution in *dop^1^* embryos is reminiscent of the droplet-transport defect in embryos mutant for the regulator Klar [33]. However, Klar expression and distribution were very similar between wild-type and *dop^1^* embryos (Figure 2I and 2K), and Klar was physically associated with lipid droplets in the mutant, as it is in the wild-type (Figure 2L). To test whether Klar function was affected in *dop^1^* embryos, we took advantage of the observation that in embryos that completely lack Halo the direction of net transport in early cycle 14 depends on Klar: it is apical in the presence of Klar but basal in the absence of Klar (unpublished data). When we abolished Halo expression pharmacologically, droplet motion was net apical in wild-type and *dop^1^* embryos but net basal in *klar* embryos (Figure S1). Thus, Klar is not only expressed but also functional in *dop^1^* embryos. Expression and function of Halo also appeared to be unaffected in *dop^1^* embryos (Figure S2).

Lipid droplets move bidirectionally along microtubules, and their apical-basal distribution results from the relative contribution of plus- and minus-end motion [33]. Altered droplet distribution in *dop^1^* embryos was not due to lack of droplet motion per se since droplets moved bidirectionally before, during, and after cellularization (unpublished data). This observation indicates that the motors driving droplet transport as well as the microtubule tracks are grossly intact. We conclude that the basic machinery responsible for droplet motion is intact and that *dop^1^* alters a specific aspect of transport resulting in faulty regulation of net transport direction.

In summary, the *dop^1^* mutation reveals the existence of a maternally established control mechanism to regulate specific events at the MBT: formation and growth of membrane at the start and during cellularization, and a developmentally regulated switch in organelle transport at the end of cellularization. Importantly, *dop^1^* does not pleiotropically impair embryogenesis per se; we did not observe developmental defects prior to the MBT. It remains to be established whether the defects in the *dop^1^* mutants reflect a common problem with microtubule-based transport or independent molecular mechanisms. Uncovering the underlying molecular mechanisms should provide new insights into the regulation of the morphogenetic events associated with the MBT.

### *dop* Is Allelic to *ago2*

**Figure 3:**
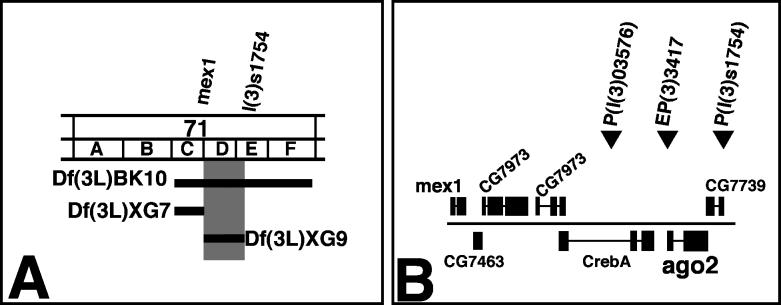
Genetic and Molecular Analysis of the *ago2^dop^* Mutations (A) Genetic characterization of the *dop* genomic region. Deletion mapping identified the cytological interval 71D1-E1 as the region uncovering the *dop* locus. Black bars represent the deleted regions. *Df(3L)BK10* represents the deletion that was used in the initial screen for female-sterile mutations by Galewsky and Schulz [25]. The distal breakpoint of *Df(3L)XG9* is to the right of the *mex1* gene (based on PCR mapping) and the proximal breakpoint is to the left of CG7739 (based on complementation of *l(3)s1754* and PCR probing for *CG7739*). (B) Genomic organization of the 71D1 to 71E3 region as predicted from the release 3 of the annotated genomic sequence by the Berkeley *Drosophila* Genome Project. The 45-kb region contains six predicted genes and three P-element-insertions. The P-insertions *P(l(3)s1754)*, *P(l(3)03576,* and *P{EP}EP3417* were utilized to produce small deletions by male recombination (unpublished data).

As a first step toward characterizing this new mechanism controlling the MBT, we sought to identify the gene affected by the *dop^1^* mutation. Using chromosomal deletions, we mapped *dop^1^* to a 45-kb genomic region that contains six predicted genes (Figure 3). P-element insertions within this interval were employed to produce small deletions by male recombination [37]. The majority of the recombinant chromosomes that failed to complement *dop^1^* were obtained with two insertions that flank the genes for *CG7739* or *ago2* (Figure 3B and unpublished data). It seemed therefore likely that *dop* is represented by one of these two genes. We generated rescue constructs in which the maternal α4-tubulin67C promoter drives cDNAs for *CG7739* or *ago2*. The *mat::tub-ago2* transgene, but not *mat::tub-CG7739*, rescued embryos derived from *dop^1^* mothers to viability (Table 1).

**Table 1:**
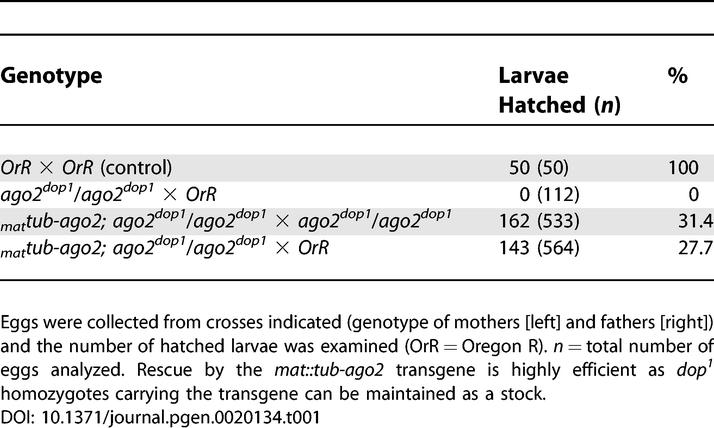
Rescue of *dop^1^* Mutants by Maternally Expressed Full-Length *ago2* cDNA

The conclusion that *dop^1^* is an allele of *ago2* is supported by an independent allele, *dop^46^*, that we created by transposase-induced reversion of *EP(3L)3417*, a P-element inserted within the 5′ UTR of *ago2*. Females homozygous or hemizygous for *dop^46^* are sterile, and their embryos can be rescued to viability by expression of the *mat::tub-ago2* transgene. Embryos derived from *dop^46^* homozygous or hemizygous females showed defects in early development very similar to embryos from *dop^1^* mothers. *dop^46^* failed to complement both the lethality and the cellularization defects of *dop^1^*. We conclude that *dop* mutations represent mutant alleles of *ago2.* In the following, these alleles will be called *ago2^dop1^* and *ago2^dop46^*, respectively. Embryos derived from homozygous or hemizygous mutant mothers will be referred to as *ago2^dop^* embryos.

The phenotype of *ago2^dop1^* strongly suggests that this mutation specifically disturbs processes at the MBT, as it did not impair syncytial development of the embryo. To test this notion, we used the *Gal4/UAS* system to drive expression of *ago2* cDNA at the onset of zygotic transcription. We found that zygotic expression of Ago2 was sufficient to rescue *ago2^dop1^* mutant embryos to viability (Table 2). We therefore conclude that the major defects in *ago2^dop^* mutant embryos are due to compromising a zygotic function and are not simply the consequences of earlier defects in oogenesis.

**Table 2:**
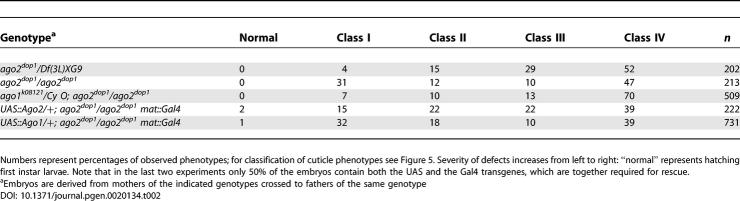
Genetic Interaction of *ago2^dop1^* with *ago1*: Distribution of Larval Cuticle Phenotypes

### *ago2^dop1^* Impairs RNAi

**Figure 4:**
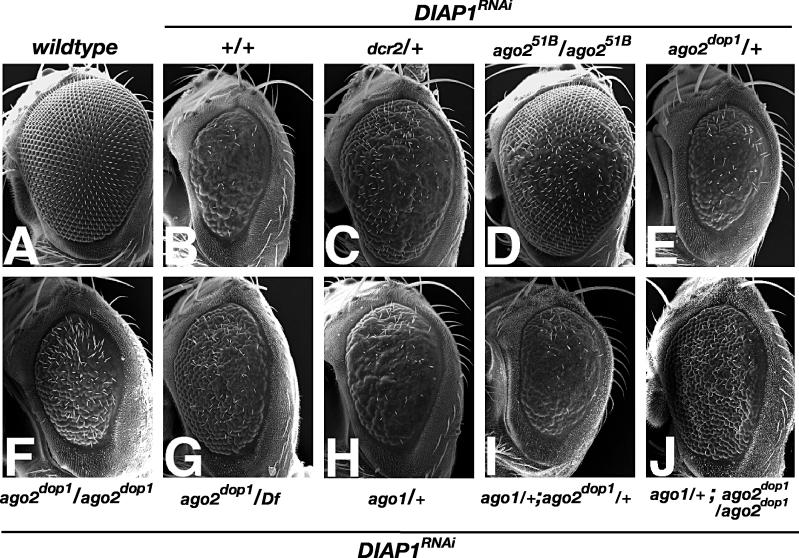
Requirement of *ago2* and *ago1* for siRNA-Mediated RNAi Effects of mutations on siRNA-mediated RNAi were assayed using a *UAS* construct that reduces the function of the cell death inhibitor DIAP1 by expression of dsRNA (*DIAP1^RNAi^*: *GMR::Gal4,UAS::DIAP1^RNAi^*) [38]. *DIAP1^RNAi^* in the compound eye leads to the loss of cells and reduces the normal size of the eye. Scanning EM micrographs of compound eyes of 2- to 4-d-old females of the following genotypes are indicated: (A) wild-type compound eye; (B) *GMR::Gal4, UAS::DIAP1^RNAi^/CyO*; (C) *GMR::Gal4, UAS::DIAP1^RNAi^*/*dcr-2**^L811fsX^***; (D) *GMR::Gal4, UAS::DIAP1^RNAi^/CyO*; *ago2^51B^*/*ago2^51B^*; (E) *GMR::Gal4, UAS::DIAP1^RNAi^/CyO*; *ago2^dop1^/TM6*; (F) *GMR::Gal4, UAS::DIAP1^RNAi^/CyO*; *ago2^dop1^/ago2^dop1^*; (G) *GMR::Gal4, UAS::DIAP1^RNAi^; ago2^dop1^/Df(3L)XG9;* (H) *GMR::Gal4, UAS::DIAP1^RNAi^/ago1^K08121^;* (I) *GMR::Gal4, UAS::DIAP1^RNA^*/*ago1^K08121^; ago2^dop1^/TM6; and* (J) *GMR::Gal4, UAS::DIAP1^RNAi^*/*ago1^K08121^; ago2^dop1^/ago2^dop1^*.

Ago2 is known to be essential for RNAi mediated through siRNAs [9,11]. Is abnormal siRNA-induced RNAi the molecular basis for the severe organismal phenotypes in the *ago2^dop^* alleles? To test whether *ago2^dop^* mutations impair RNAi, we employed *DIAP1^RNAi^*, a double-stranded RNA (dsRNA) construct against the cellular caspase inhibitor DIAP1 [38]. When this construct is expressed in the developing eye, DIAP1 levels are reduced, causing cells to die by apoptosis; as a consequence, the eyes are significantly smaller (Figure 4A and 4B).

In this experimental system, the efficiency of RNAi induced by long dsRNA can be assessed by the size of the eye: efficient RNAi results in severely reduced eyes; complete disruption of RNAi leads to normal-sized eyes. In flies with only a single copy of the *Dcr-2* gene, this phenotype was partially suppressed (Figure 4C), confirming that the activity of *DIAP1^RNAi^* depends on siRNA production through Dcr-2. We also tested a reported null allele of *ago*2 in this assay: *ago2^51B^* has a deletion of exons 1 and 2 and completely abolishes siRNA-directed RNAi in embryo extracts [11]. In flies homozygous for *ago2^51B^*, the *DIAP1^RNAi^* phenotype was completely suppressed (Figure 4D). Together these results are consistent with the biochemical requirements of Dcr-2 and Ago2 for RNAi [11,24].

The *ago2^dop1^* mutation impaired RNAi only partially. Animals heterozygous for *ago2^dop1^* did not exhibit a significant modification of the *DIAP1^RNAi^* phenotype (Figure 4E). We observed mild suppression in *ago2^dop1^* homozygotes and stronger suppression in *ago2^dop1^* hemizygotes, but even in the most extreme cases suppression was less pronounced than for *ago2^51B^* (Figure 4F and 4G). Thus, the *ago2^dop1^* mutation compromises, but does not abolish, Ago2′s function to mediate RNAi induced by long dsRNA.

When we examined *ago2^51B^* mutant embryos, in which RNAi is completely abolished, the embryos appeared largely normal morphologically, consistent with previous reports on the effect of another *ago2* null allele, *ago2^414^* [11,39]. As described recently, a minor fraction of the embryos derived from mothers homozygous for *ago2^51B^* (44% of embryos, *n* = 786) or *ago2^414^* (33% of embryos, *n* = 200) exhibit defects during syncytial cleavages [40]. Embryos displaying syncytial defects were grossly abnormal and did not develop beyond that stage. All other embryos obtained from *ago2^51B^* or *ago2^414^* homozygous mothers developed normally, and cellularization of these embryos was indistinguishable from the wild-type (Figure S3). We also analyzed the distribution of lipid droplets in *ago2^51B^* homozygous embryos and found no defects in net transport of droplets (Figure S4). Thus, it seems unlikely that the defects in membrane growth and microtubule transport in *ago2^dop1^* embryos are due to a failure in the siRNA pathway.

This conclusion is further supported by the lack of morphogenesis defects in *Dcr-2* mutants. Lee et al. [24] reported that mutations in *Dcr-2* are viable, and we found that embryos derived from *Dcr-2* homozygotes exhibit normal cellularization and net lipid-droplet transport (unpublished data). We therefore conclude that *ago2^dop^* mutations alter the activity of the *ago2* gene and that the forms of Ago2 encoded by the *ago2^dop^* alleles might interfere with a pathway distinct from siRNA-triggered RNAi.

### *ago2* Interacts Genetically with *ago1*

One possibility to explain the early embryonic phenotype of *ago2^dop^* mutants is that mutations in one Argonaute family member might affect the function of another. A good candidate for this other Argonaute gene is *ago1*. While biochemical studies have concluded that Ago1 is dispensable for siRNA-triggered RNAi [11], it has also been reported that mutations in *ago1* mildly suppress siRNA-triggered RNAi in the embryo [21]. Consistent with the latter report, we found, using the *DIAP1^RNAi^* reporter construct, that reduction of *ago1* gene dosage mildly suppresses the RNAi response in the compound eye (Figure 4H). This suppression can even be enhanced by *ago2^dop1^*; flies that are heterozygous for *ago1* and homozygous for *ago2^dop1^* exhibit a stronger suppression of the *DIAP1^RNAi^* phenotype as either mutation alone (Figure 4I and 4J). We conclude that *ago1*, at least in the eye, is required for siRNA-mediated RNAi and thus Ago1 and Ago2 might have partially overlapping functions.

**Figure 5:**
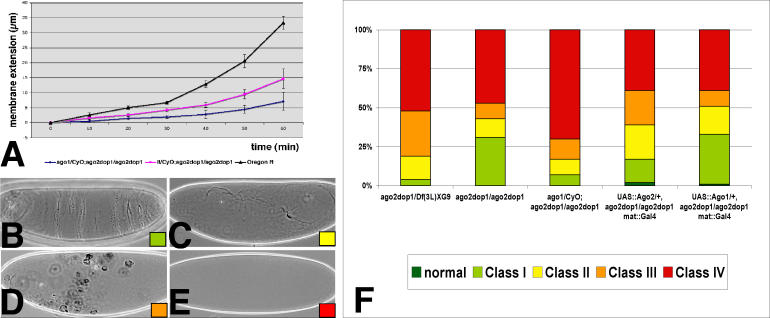
Genetic Interaction of *ago2^dop1^* with *ago1* Reduction of the *ago1* gene doses enhances the *ago2^dop1^* cellularization phenotype. (A) Kinetics of membrane extension during cellularization in embryos derived from *ago1^K08121^*/CyO; *ago2^dop1^*/*ago2^dop1^* females. Embryos from *Kr^If^/CyO*; *ago2^dop1^*/*ago2^dop1^* display the characteristic *dop* delay in membrane growth during cellularization (purple line; *n* = 9) relative to the wild-type (Oregon R; black line; *n* = 6). Embryos from *ago1^K08121^*/CyO ; *ago2^dop1^*/*ago2^dop1^* mutants (blue line; *n* = 9) show a strongly reduced furrow progression even during fast phase. (B–E) Embryos derived from mothers homozygous or hemizygous for *ago2^dop1^* do not hatch and produce abnormal larval cuticles, which can be grouped into four classes. (B) Class I (continuous cuticle): such embryos form a continuous cuticle with more or less severely affected denticle belts. (C) Class II (shield): such embryos produce a shield of continuous cuticle, reminiscent of neurogenic mutants. (D) Class III (*crumbs*-like): such embryos produce only small globular remnants of cuticle, reminiscent of mutations in genes required for epithelial polarity, such as *crumbs*. (E) Class IV (no cuticle): embryos in this class did not produce any cuticle at all. (F) Graphic presentation of the distribution of cuticle phenotypes of the different classes; color codes are indicated on (B–E). Original data are presented in Table 2.

Given the interaction of *ago1* and *ago2* in siRNA-mediated silencing, it is conceivable that these two genes also have overlapping roles at the MBT. Indeed, reduction of *ago1* function enhanced the cellularization phenotype of *ago2^dop1^* homozygotes: membrane growth was strongly compromised, and only very little membrane extension was observed (Figure 5A). This enhanced defect in cell formation was also evident from a more severe disruption of larval cuticle formation (Figure 5B–5F and Table 2). Remarkably, zygotic expression of *UAS::ago1* was sufficient to ameliorate the cuticle defects of *ago2^dop1^* mutant embryos and to allow some of them to develop into adult flies (Figure 5F and Table 2). Thus, the severity of the *ago2^dop1^* phenotypes is sensitive to the levels of expression of *ago1*, suggesting that in *ago2^dop1^* mutants Ago1 function has become limiting.

**Figure 6:**
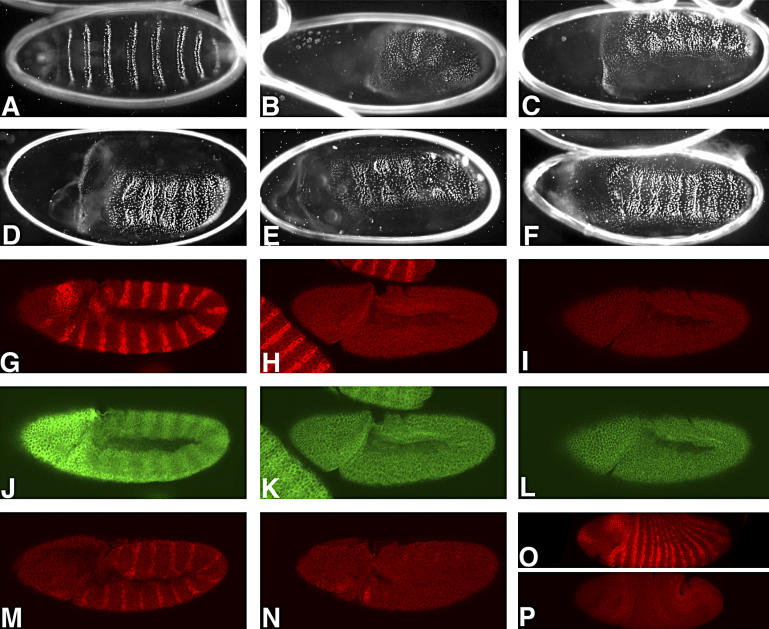
Requirements of *ago1*, *ago2,* and *Dcr-1* for Segmentation Segment-polarity defects in zygotic *ago1*, *ago2* homozygous mutant embryos are evident in alterations of the larval cuticle (A–F) or the pattern of immunolabeling (G–O). All embryos are oriented with their anterior to the left. (A–F) Parents of the indicated genotypes were mated and the cuticles of those embryos that failed to hatch were examined: (A) Embryos double mutant for *dcr-2**^L811fsX^*** and *dcr-1^Q1147X^* do not hatch, but exhibit normal cuticle differentiation. (B) Class I cuticles from *ago1^K08121^/CyO; ago2^dop1^/ago2^dop1^* mutant parents exhibit a lawn of denticles, indicative of a defect in establishing segment polarity. (C) Embryos from *ago1^K08121^/CyO; ago2^dop1^/TM6* mutant parents develop a continuous cuticle with an anterior hole and a strong segment polarity defect. (D) Embryos from *ago1^K08121^/CyO; ago2^51B^/TM6* mutant parents or from (E) *ago1^K08121^/CyO; ago2^51B^/ago2^51B^* parents show similar segment polarity defects. (F) Embryos obtained from *ago1^K08121^/CyO; dcr-1^Q1147X^/TM6* parents also exhibit a typical segment polarity defect. (G–N) Immunolabeling of Wg (G–I), Arm (J–L), or En (M, N), proteins in extended germband embryos (stage 9). To identify homozygous embryos, *CyO[hb::lacZ]* and *TM3[hb::lacZ]* balancer chromosomes were used. The presence of the *[hb::lacZ]* transgene on the balancers results in ß-galactosidase expression in the anterior of the embryo (J). (G, J, M) Embryos heterozygous for *ago1^K08121^; ago2^dop1^*. Wg and En proteins are expressed in 14 stripes; cytoplasmic Arm protein is elevated in 14 stripes (J). (H, K, L) In *ago1^K08121^; ago2^51B^* zygotic double mutants, Wg expression is completely abolished (H). Cytoplasmic levels of Arm (K) are equally low in all cells of the embryo and expression of En (N) is not maintained and fades from stage 9 onward. (I, L) Homozygous *ago1^K08121^; dcr-1^Q1147X^* double mutant embryos also lack expression of Wg (I) and hence also fail to accumulate Arm (L) stripes. (O, P) Gastrula stage *ago1^K08121^; dcr-1^Q1147X^* double mutant embryo (stage 7) (P) lacks detectable Wg protein, while Wg is expressed in heterozygous sibling embryos (O).

Since the overlapping functions of Ago1 and Ago2 were detected using our *ago2^dop^* alleles, the question arises of whether this genetic interaction is an unusual feature of these particular alleles or whether the two genes cooperate more generally. We were able to distinguish between these possibilities when we discovered a novel phenotype of *ago1* and *ago2* in larval cuticle formation (Figure 6). Embryos homozygous for both *ago1^k08121^* and *ago2^dop1^* displayed cuticles with characteristic patterning defects, similar to those described for mutations in the *wingless (wg)* or *arm* genes (Figure 6B and 6C). This strong segment-polarity defect was also observed in embryos double mutant for the two loss-of-function alleles *ago1^k08121^* and *ago2^51B^* (Figure 6D and 6E). Because such defects are never observed in single mutants for *ago1* or *ago2* ([20]; W. J. Meyer and H. A. J. Müller, unpublished data), these data indicate that *ago1* and *ago2* act in a partially redundant fashion. In addition, these observations reveal a previously unrecognized essential role of Argonaute genes in segmental patterning.

To identify the molecular basis for this patterning defect, we examined the expression of Wg, Engrailed (En), and Arm proteins in the double mutants. In the wild-type, Wg and En are initially expressed in 14 nonoverlapping segmental stripes (Figure 6G and 6M). In response to Wg signaling, levels of cytoplasmic Arm increase and En expression is maintained in the receiving cells (Figure 6J; [41]). In embryos zygotically homozygous for both *ago1^k08121^* and *ago2^51B^,* Wg protein was not detected in extended germ-band stages (Figure 6H). The lack of expression of Wg protein in these embryos is sufficient to result in the observed patterning defects. As a consequence of the lack of Wg protein expression, cytoplasmic Arm stripes are not present and En expression is not maintained in these embryos (Figure 6K and 6N). This genetic interaction between *ago1* and *ago2* alleles indicates a partially redundant function of the two Argonaute family members in the positive regulation of Wg protein expression in the early embryo.

Since Ago1 is an essential component of the miRNA-triggered RNA silencing, we reasoned that the observed partial redundancy with Ago2 might reflect a function of Ago2 in the miRNA pathway. If this assumption were correct, *ago1* might exhibit a similar genetic interaction with mutations in other crucial components of the miRNA pathway. The best candidate to test for this interaction is Dcr-1, because Dcr-1 is essential for the processing of pre-miRNAs [24]. Indeed, we found that double mutants of the loss-of-function alleles *ago1^k08121^* and *Dcr-1^Q1147X^* exhibit the same segment-polarity cuticle phenotype and lack of Wg expression as *ago1, ago2* double mutants (Figure 6F, 6I, and 6L). In *ago1*, *Dcr-1* double mutants, Wg protein was not detected even in gastrula stages (Figure 6O and 6P). Embryos singly mutant for *Dcr-1^Q1147X^* or double mutant for *Dcr-2**^L811fsX^*** and *Dcr-1^Q1147X^* do not show such defects (Figure 6A and unpublished data). Because a reduction in the zygotic expression of two major components of the miRNA pathway can result in segment polarity defects, these data suggest an as-of-yet unrecognized requirement of miRNA function for segmentation and the regulation of Wg expression.

In summary, our genetic analysis has uncovered several instances in which Ago1 and Ago2 appear to act in a partially redundant and partially overlapping manner: in siRNA-mediated RNA interference, in the regulation of Wg expression in the early embryo, and for morphological changes associated with the MBT.

### Biochemical Interaction of Ago1 and Ago2

**Figure 7:**
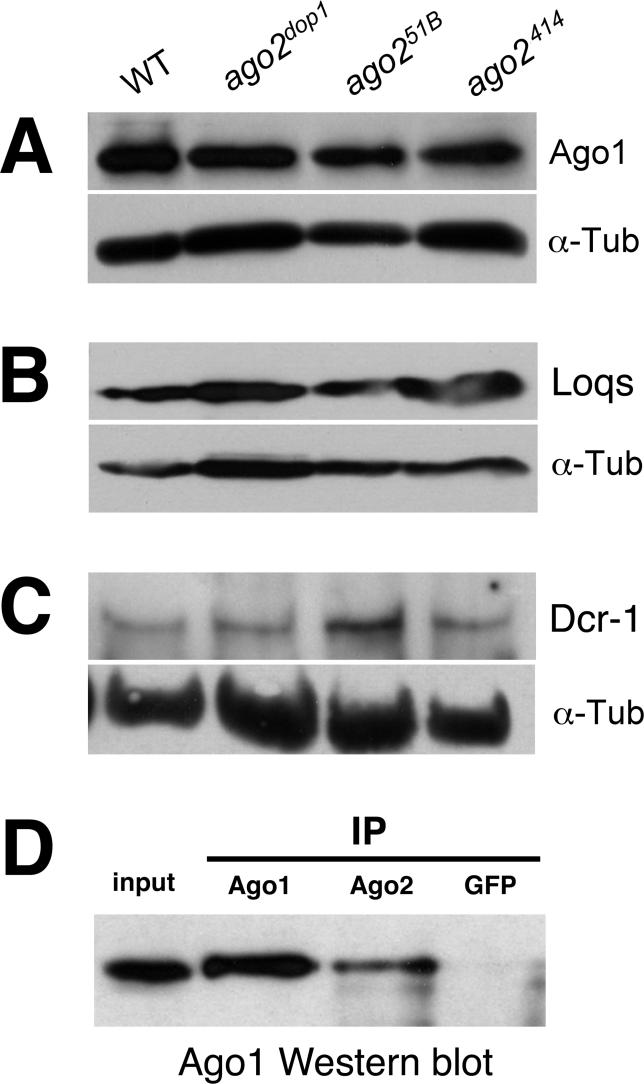
Analysis of Protein Levels of Ago1, Loqs, and Dcr-1 in *ago2* Mutants and Coprecipitation of Ago1 and Ago2 (A) Western blot using anti Ago1 antibodies. Ago1 protein level in embryo extracts was very similar in the wild-type (*w^1118^*), *ago2^dop1^*, *ago2^51B^*, and *ago2^414^* mutants; α-tubulin was used as a loading control. Likewise, the protein levels of Loqs (B) and Dcr-1 (C) are largely unimpaired in extracts from *ago2^dop1^*, *ago2^51B^*, and *ago2^414^* mutants when compared to the wild-type (*w^1118^*). In (C) ovary extracts were used instead of embryo extracts. (D) Coimmunoprecipitation experiments of Ago1 with Ago2. Ago1 protein in extracts from wild-type embryos is shown in the left lane (input). Immunoprecipitations (IP) were performed using anti Ago1 and Ago2^Cterm^ antibodies or with an antibody against GFP as a control; immunoprecipitates were analyzed by Western blotting with Ago1 antibodies. All experiments were carried out several times with essentially the same results and representative blots are depicted. A semiquantitative analysis of several independent Western blots is shown in Figure S7.

What is the molecular basis for the observed genetic interaction between *ago1* and *ago2?* It is conceivable that depletion of Ago2 leads to a reduction in the levels of Ago1 or shared components of the RISC assembly machinery. To investigate this possibility, we analyzed Ago1 protein levels in *ago2* mutants by Western blotting. We find that Ago1 protein levels are wild-type in embryos derived from *ago2^dop1^* homozygotes, as well as from *ago2^51B^* or *ago2^414^* homozygotes (Figure 7A). We also tested the levels of Dcr-1 and Locquacious (Loqs), a dsRNA binding protein that can be part of a protein complex with Ago1 and Dcr-1 and is important for the function of Dcr-1 to process pre-miRNAs [42,43]. Like in the case of Ago1, Loqs and Dcr-1 protein levels were largely unimpaired in *ago2^dop1^*, *ago2^51B^*, or *ago2^414^* homozygotes (Figure 7B and 7C). We therefore conclude that mutations in *ago2* do not result in global changes of the protein levels of Ago1, or shared components involved in RISC assembly.

Our genetic data demonstrate that Ago1 and Ago2 act in a partially redundant fashion in siRNA-triggered RNAi as well as during morphogenesis and pattern formation in the embryo. One possible explanation for this interaction would be that Ago1 and Ago2 have overlapping functions as part of a common protein complex. To examine this possibility, we used Ago2 antibodies to immunoprecipitate Ago2 protein complexes from embryo lysates and assayed for the presence of Ago1. We found that anti-Ago2 antibodies coprecipitate Ago1 protein from these extracts (Figure 7D). These results indicate that subpopulations of Ago1 and Ago2 proteins are present in a common complex, consistent with the idea that Ago1 and Ago2 have overlapping functions.

### *ago2^dop^* Mutations Affect Glutamine-Rich Repeats in the Amino-Terminus of Ago2

**Figure 8:**
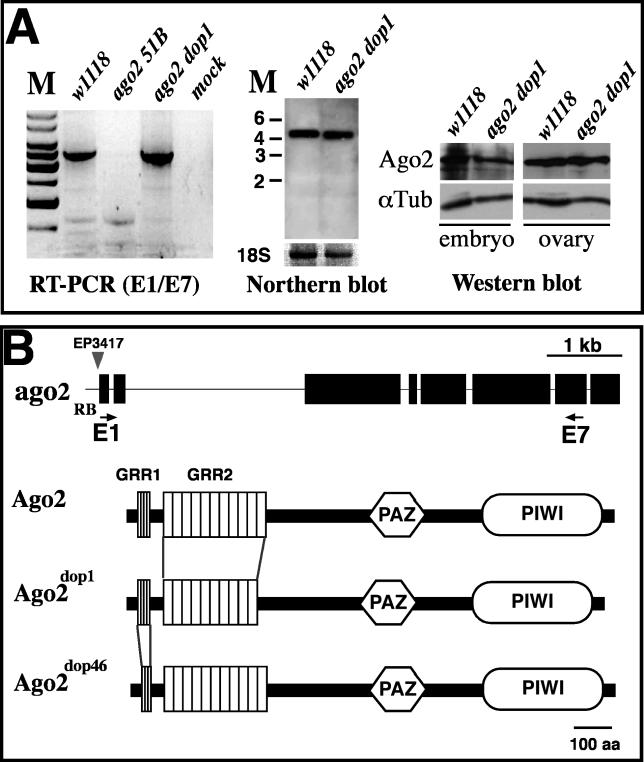
Molecular Analysis of *ago2^dop^* Mutations (A) RT-PCR and Northern blot analyses of *ago2* transcripts. For RT-PCR, we used a forward primer in exon1 (E1) and a reverse primer in exon 7 (E7) as indicated in (B). This combination revealed a transcript fragment of 3,488 nucleotides (right hand panel), which is present in *w^1118^* (wild-type) and *ago2^dop1^*, but absent in *ago2^51B^*; note the slightly faster mobility of the E1/E7 amplicon in *ago2^dop1^* mutants. Northern blot using poly-A**^+^** RNA prepared from *w^1118^* or *ago2^dop1^* mutant embryos. A transcript of about 4 kb is visible; note that the *ago2^dop1^* transcript runs slightly faster on the gel. The amount of 18S ribosomal RNA was used as a loading control. Levels of *ago2* transcript were very similar in wild-type and *ago2^dop1^*. Protein levels of Ago2 were determined by Western blot using anti-Ago2 antibodies. In extracts from embryos or ovaries levels of Ago2 protein were largely unimpaired in *ago2^dop1^* mutants compared to controls (*w^1118^*). (B) Genomic structure of *ago2* and mutations in *ago2^dop1^* and *ago2^dop46^*. The *ago2* gene spans 6,930 nucleotides of genomic DNA and contains eight exons (RB-transcript; an alternatively spliced form, called RC encodes for a very similar protein and is not indicated in this cartoon). Cartoon of Ago2 protein indicates the positions of the GRR region at the amino-terminus, a central PAZ domain, and a carboxyl-terminal PIWI domain*.* The amino-terminus of Ago2 contains four imperfect GRR1 (LQQPQQ) repeats and 11 imperfect GRR2 (QGGHQQGRQGQEGGYQQRPPGQQ) repeats. The RB transcript codes for a protein of 1,214 aa. The protein encoded by the *ago2^dop1^* allele contains only 1,191 aa and lacks one of the GRR2 repeats (repeat number 9 or 10). The *ago2^dop46^* mutation represents an 18-nucleotide deletion, leading to the loss of one GRR1 and a predicted protein of 1,208 aa.

Our data on the *ago2^dop^* alleles indicated that these mutations compromise Ago2′s function in the siRNA pathway and might also disturb the normal function of Ago2 such that it negatively interferes with Ago1 function. This suggests that a molecular characterization of the *ago2^dop^* mutations should give insight into the mechanism by which Ago2 and Ago1 interact. Our molecular analysis indicates an important role for the previously uncharacterized amino-terminal GRR domain (Figure 8). This domain is rich in glutamines and is largely composed of four imperfect copies of the 6–amino acid (aa) repeat GRR1 and 11 imperfect copies of the 23-aa repeat GRR2 (Figures 8B and S5).

When we compared *ago2* expression by RT-PCR or Northern blotting, we found that *ago2* mRNA was expressed in *ago2^dop1^* but that its size was slightly shorter than in the wild-type (Figure 8A). Importantly, despite the size difference, the total levels of *ago2* transcript are the same in the wild-type and *ago2^dop1^* mutants (Figure 8A). We also assessed whether the *ago2^dop1^* mutation affects the total protein levels of Ago2 and found that Ago2 protein levels are unimpaired in the mutant (Figure 8A). Thus, the *ago2^dop^* mutation does not affect the normal accumulation of Ago2 mRNA or protein in the embryo.

We found that the genomic sequence of *ago2^dop1^* exhibits a 69-nucleotide in-frame deletion in exon 3 that leads to the loss of exactly one copy of GRR2 (Figure 8B). The molecular lesion in *ago2^dop46^* is an 18-nucleotide in-frame deletion in exon 2, which results in the loss of one copy of GRR1 (Figure 8B). DNA sequence analysis did not reveal any other mutations in the coding region of the *ago2^dop^* alleles. These data provide the first evidence that the amino-terminal domain of Ago2 is essential for the normal function of the protein and that mutations in the GRRs reduce Ago2′s activity in siRNA-triggered RNAi. Strikingly, even a slight reduction in the number of either type of GRRs has severe biological consequences. Based on the genetic interactions of *ago2^dop^* and *ago1* and the fact that Ago1 and Ago2 are part of the same protein complex, we propose that these mutations in the GRR domain result in aberrant Ago2 variants that reduce Ago2 activity and might also negatively interfere with Ago1-dependent processes.

### Amino-Terminal Glutamine-Rich Domains Are a Conserved Feature of Insect Ago2 Proteins

Evolutionary comparisons suggest that this mechanism of Ago2 regulation is not restricted to Drosophila melanogaster. In *D. melanogaster,* exon 3 of *ago2* encodes the major portion of the GRR domain (123 glutamates in a 333-aa stretch, including all copies of GRR2) plus an 89-aa region conserved in other Argonaute family members from both animals and plants. We identified the likely corresponding exons in the genomic sequences of six additional *Drosophila* species and found the same bipartite structure (Table 3 and Figure S5A): an amino-terminal glutamine-rich region followed by the conserved carboxyl-terminal stretch (58% to 90% sequence similarity). Similar amino-terminal glutamine-rich domains of Ago2 are predicted from the genomic sequences of two mosquito species and of honeybees (Figure S5B). Although we have not yet examined expression outside *D. melanogaster,* both exon prediction algorithms and cDNA evidence from D. simulans and A. gambiae suggest that these glutamine-rich regions are indeed expressed (see Materials and Methods).

**Table 3:**
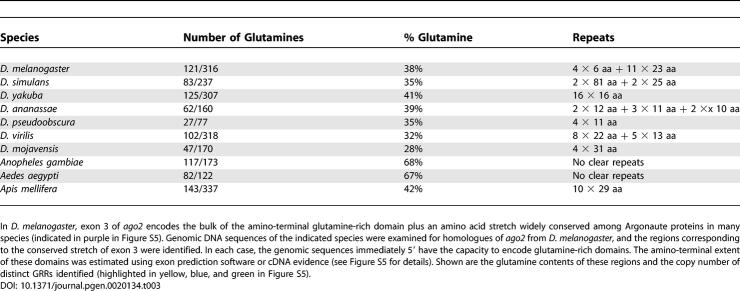
Amino-Terminal Glutamine-Rich Domains in Predicted Ago2 Orthologs of Insect Species

Within a single species, these glutamine-rich amino-termini typically contain multiple imperfect copies of one to three repeat units. Yet between species, the sequences are highly variable. This observation suggests that the primary sequence of this region evolves rapidly, presumably by repeat expansion and contraction [44]. Despite this variation, the glutamine-rich character of this region is preserved in all the species examined. This pattern is reminiscent of the yeast prion Sup35: across yeast species, the primary sequence of the amino-terminal prion-determining domain evolved rapidly but retained high glutamine content [45,46]. In many proteins, glutamine-rich regions can mediate protein aggregation, and the propensity to aggregate increases with increasing GRR copy number [47,48]. Therefore this domain of Ago2 may similarly control the aggregation or intracellular distribution of the protein.

## Discussion

In *Drosophila,* two major molecular pathways of RNA silencing have recently been defined: miRNA-induced silencing and siRNA-induced RNAi [1]. At the level of Argonaute family members, Ago1 has been implicated in miRNA function while Ago2 was shown to be essential for siRNA function. Our analysis provides genetic and biochemical evidence that Ago1 and Ago2 have overlapping functions both in siRNA-triggered RNAi and during early embryogenesis.

We found that, in addition to the PAZ and PIWI domains conserved in all family members, insect orthologs of Ago2 contain an amino-terminal GRR domain. The *ago2^dop^* alleles allow us to probe the function of this domain. Even the subtle alterations in these alleles have striking organismal phenotypes, but the absence of Ago2 (in the reported null alleles) does not. While the mutant Ago2 proteins still support siRNA function to some extent, they also interfere with Ago1-dependent processes.

### A Function for the Amino-Terminal Glutamine-Rich Repeats of Ago2

In other proteins, glutamine-rich domains have been implicated in protein aggregation, such as in certain neurodegenerative diseases that involve the formation of long-lived protein aggregates (e.g., the PolyQ domain of mutant Huntingtin). Extension of the glutamine-rich region promotes aggregation, and the length of the polyglutamine extension correlates with the severity of the disease [22]. Glutamine-rich domains are also involved in the mechanism by which yeast prions switch between soluble and aggregated states [49,50]. For the translation factor Sup35, e.g., increases in the copy number of GRRs in the prion domain favor the aggregated, inactive state; decreases in the copy number favor the soluble, active state [47,51]. Our genetic and molecular analyses of the *ago2^dop^* alleles thus raise the tantalizing possibility that the GRRs regulate Ago2 by modulating its aggregation state. Unlike in the polyglutamine diseases, however, it is the reduction, rather than the expansion, of the GRR region that leads to an aberrant Ago2 protein. *Drosophila* Ago2 may therefore provide a unique inroad for dissecting the normal organismal function of glutamine-rich or PolyQ domains.

Since Ago2 is an essential component of protein complexes, such as the RISC, control of its aggregation state is conceivably important for its function. Mammalian Argonaute proteins are localized to GW bodies, cytoplasmic compartments analogous to yeast P-bodies, which are centers of mRNA degradation [52,53]. Central components of GW bodies, like GW182 and decapping enzymes DCP1:DCP2, have recently been shown to also be involved in miRNA-mediated gene silencing in *Drosophila* cultured cells [54]. The presence of both Ago1 and Ago2 in GW bodies is consistent with our biochemical studies. An important next step for unraveling the molecular function of the Ago2 GRR domain will be to determine whether the *ago2^dop^* alleles alter the recruitment of Ago2 to particular cytoplasmic mRNA degradation complexes. Such recruitment via glutamine-rich domains need not necessarily inactivate the protein: in the translation factor CPEB from *Aplysia*, a glutamine-rich prion-like amino-terminal domain promotes protein aggregation, and it is the aggregated form that has the greatest capacity to stimulate translation [55].

### Specificities of Ago1 and Ago2 Functions

Previous analyses had suggested a simple model of division of labor between Argonaute proteins in *Drosophila,* with Ago1 specific for miRNA-directed silencing and Ago2 involved in siRNA-triggered RNAi. However, our genetic data add to emerging evidence that these proteins play much broader roles. Ago2, for example, appears to have functions beyond siRNA-induced RNAi. It has been proposed that in larval neurons Ago2 is recruited via the dFMR1 protein to certain RNP complexes, including those containing the *PPK1* mRNA. This recruitment is functionally important since in the *ago2^51B^* allele *PPK1* mRNA levels are not properly downregulated; thus, Ago2 may play a role in the turnover of specific transcripts [39].

For Ago1, on the other hand, it is well established that it has a function in miRNA-directed RNA silencing. But while in biochemical assays Ago1 is not essential for siRNA function, *ago1* mutations impair the response of siRNA-triggered RNAi in vivo ([11,21]; the present work). Our data provide further evidence for overlapping functions of Ago2 and Ago1 in siRNA-directed RNAi. It is possible that although Ago2 is in principle sufficient to promote siRNA-directed RNA decay, in vivo the two proteins act in concert to make this process more efficient.

It is unlikely that the morphogenesis phenotypes of *ago2^dop^* mutant embryos are simply caused by disturbing the function of Ago2 in RNAi. Unlike *ago2^dop1^* mutants, *ago2* alleles that completely abolish experimental siRNA-induced responses do not cause these gross morphological defects and exhibit problems with nuclear migration only during syncytial stages; these phenotypes occur with a moderate penetrance such that animals homozygous for these alleles can be kept as a stock [11,24,39,40]. Rather, our genetic data suggest that *ago2^dop^* mutations compromise the function of both Ago2 and Ago1 in controlling specific aspects of the MBT. A genome-wide analysis of mRNA targets regulated by Argonaute proteins has recently shown that Ago1 and Ago2 are required for the regulation of a common set of miRNA targets, despite the fact that only Ago1 is essential for miRNA function in vitro [56]. In S2 cells, both Ago1 and Ago2 coprecipitate with specific miRNAs, suggesting that not only Ago1, but also Ago2, is able to bind miRNAs [56]. Based on our results, it is conceivable that the interaction of miRNAs with Ago2 is indirect, namely that Ago2 coprecipitates those miRNAs that are bound to Ago1. While the exact mechanisms need to be resolved, the available data provide ample support for our conclusion that Ago1 and Ago2 act in a partially redundant fashion during early embryogenesis.

It is conceivable that the *ago2^dop^* mutations not only interfere with Ago1 and Ago2 function but might affect a common factor that is essential for both Ago1 and Ago2 or for Argonaute protein function in general. Preliminary observations suggest that mutations in other Argonaute family members, *piwi* or *aubergine,* might also interact genetically with *ago2^dop^* alleles (unpublished data). We currently favor the model that disrupting both Ago1 and Ago2 function is sufficient to cause the observed defects at the MBT because *ago2^dop1^* mutants can be rescued by zygotic expression of either *ago1* or *ago2*. A test of this notion will be to determine the phenotypic consequences for embryos when both the maternal and zygotic expression of *ago1* and *ago2* has been eliminated. In addition, the interactions of *ago2^dop^* alleles with other components of RNA silencing pathways should be examined to further understand the genetic and molecular basis for the altered activity of Ago2^dop^ proteins during the MBT.

### Function of Argonaute Proteins in Segment Polarity

Mutations in *ago1* were originally discovered in a genetic screen for modifiers of the Wg pathway [20]. Overexpression of *ago1* rescued a defect in Wg signaling induced by depletion of cytoplasmic Arm in the wing imaginal disc. However, because embryos homozygous for a loss-of-function mutation in *ago1* did not exhibit defects in segment polarity [20], the relevance of Ago1 for normal Wg signaling remained unclear. The data presented in this paper now provide an explanation for this result. By combining loss-of-function mutations in both *ago1* and *ago2,* we demonstrate that the two Argonaute genes have partially overlapping functions and together are required for establishing segment polarity.

The function of Ago1 and Ago2 for the initial expression of Wg protein is striking. No other genes have been identified that are similarly essential for the general expression of Wg. We propose two possible explanations for this result. Ago1 and Ago2 might act to eliminate a general repressor of *wg* transcription or translation. In this case, it is conceivable that specific miRNAs exist that modulate *wg* expression by negatively regulating a repressive mechanism. Alternatively, Ago1 and Ago2 might be part of RNPs that contain *wg* mRNA, and the reduction in Argonaute function might interfere with the microtubule motor-driven localization of the transcripts. It is well established that compromising the apical localization of *wg* mRNA strongly affects the intracellular distribution and the signaling activity of the protein [57,58]. A detailed analysis of the expression and the localization of *wg* transcripts will be required to discriminate between these possibilities.

Although we have not been able to find direct evidence that any of the *ago2* alleles interfere with miRNA function in vivo or in vitro, it is interesting to note that *ago1*, *Dcr-1* double mutants exhibit the same segment polarity phenotypes as *ago1*, *ago2* double mutants. This result further strengthens the notion that in the embryo Ago1 and Ago2 might both be important for miRNA function (see above). When we employed an eye reporter assay to test if *ago2^dop^* alleles interfere with the function of the *bantam* miRNA, we failed to detect interactions (Figure S6). This result might be due to the observed redundancy of Ago2 with Ago1 function; such a redundancy was recently described for S2 cells ([56]; Figure S6). Future studies to identify the miRNAs involved and their targets might yield novel insight into the regulation of Wg expression.

An alternative explanation is that our analysis has uncovered a novel function of Argonaute protein family members. Intriguingly, the study of Kataoka et al. [20] showed that ectopically expressed Ago1 constructs could suppress Wg pathway defects even if they lacked a functional PIWI domain. This result may suggest that Ago1 function in Wg signaling does not involve its PIWI domain, hinting at an uncharacterized biochemical property of Ago1. Although too little is known at this point to speculate what such a new function might entail, it is interesting to note that there are intriguing connections between microtubules and the RNA silencing machinery: Armitage, a putative helicase required to assemble Ago2-containing RISC [59,60], is associated with microtubules in developing oocytes; the *dop* alleles of Ago2 interfere with microtubule-based processes at the MBT; and it is conceivable that Ago1 and Ago2 control the microtubule-dependent localization of *wg* mRNA. Whether or not these phenomena are explained by a shared molecular mechanism remains to be established.

In summary, the genetic interactions described in this paper are not easily reconciled with the model that different pathways in gene silencing are strictly separated. Rather, our data suggest that in the living organism these pathways, or at least crucial components of these pathways, might act in concert. Our observation that *ago1* and *ago2* cooperate in Wg signaling provides a powerful new tool to resolve some of these issues since now the function of these Argonaute proteins can be assessed using a clearly defined phenotype of a well-characterized signaling pathway.

### Function of Ago1 and Ago2 for the Midblastula Transition

Freshly laid *Drosophila* embryos contain large amounts of maternally supplied mRNAs that encode proteins essential for the earliest stages of embryogenesis [61]. As development proceeds, these maternally supplied transcripts need to be replaced by transcripts synthesized by the zygote. This process is a hallmark of the MBT. Maternal transcripts are degraded via two pathways: a maternal pathway switched on at egg activation, and a zygotic pathway activated at the MBT [62]. Our genetic analysis has shown that although *ago2^dop^* alleles represent maternal-effect mutations, they specifically perturb processes shortly after the onset of zygotic transcription at the MBT. We therefore propose that Ago1 and Ago2 are key mediators of the zygotic pathway of maternal transcript degradation. Precedence for such a scenario has recently been provided by the identification of the miR-430 miRNA family in zebrafish. miR-430 expression is strongly upregulated at the MBT and is required to specifically downregulate a set of maternal mRNAs [63]. Conversely, embryos deficient for Dicer activity display defects shortly after the MBT [63]. It remains to be determined whether miRNAs are also required for maternal transcript degradation in *Drosophila*.

The known functions and structural features of Argonaute proteins suggest a model for the underlying molecular mechanisms. It is well established that Argonaute proteins can act as ribonucleases and provide slicer activity in RISC [14,17,19,64]. During early development, Ago2 and Ago1 might act as ribonucleases that cleave maternal transcripts at the MBT. Abnormal persistence of maternal mRNAs could then interfere with the morphogenetic events usually triggered by zygotic transcription, such as membrane growth during cellularization and correct directionality of lipid-droplet transport. Alternatively, Argonaute proteins might regulate the translation of such maternal or zygotic transcripts. As we have not detected significant changes in the expression pattern of known regulators of membrane growth and droplet transport (Halo, Slam, Klar), the relevant targets are likely novel components of these regulatory pathways. Identifying them should not only give insight into the regulation of these fundamental cell-biological processes but will also shed light on the mechanisms by which the Argonaute proteins Ago1 and Ago2 work together to control developmental events.

## Materials and Methods

### *Drosophila* genetics.

Flies were kept on standard medium. The following stocks were used: *w^1118^*; *dop^1^ red e/TM6* [25]; *Df(3L)XG9/TM3*; *Df(3L)XG7/TM3*; *Df(3L)Ly,BK10/TM3*; *P{lacW}l(3)s1754/TM3*; *P{EP}AGO2^EP3417^*; *P{PZ}CrebA^03576^; Ki, P{Delta2–3}99B*; *H{PDelta2–3}HoP2.1,CyO/Bc*; *P{GawB}ptc^559.1^*; *ago2^51B^* [39]; *ago2^414^* [11]; *y w P{hsFLP}; P{neoFRT}82B; Dcr-1^Q1147X^/TM3*; *Dcr-2**^L811fsX/^**CyO* [24]; *y w P{hsFLP}*; Δ(*halo*) [35]; *klar^B^* [36]; *ago1^k08121^/CyO; UAS::Ago1* [20]; *GMR:Gal4; UAS::DIAP1^RNAi^* [38]; and *GMR:Gal4; GMR::hid; ban^EP3622^*. *ago2^dop46^* was produced by imprecise excision of *EP(3)3417*. Male recombination experiments were done according to [65]. Egg collection and cuticle preparations were according to standard methods.

### In vivo observations and immunohistology.

For in vivo observation, embryos were collected, staged on yeasted apple juice plates, and mounted in halocarbon oil 27 (Sigma-Aldrich, St. Louis, Missouri, United States) on microscope slides. Videomicroscopy was performed on a Carl Zeiss (Jena) microscope equipped with Nomarski optics, and time-lapse videos were taken on conventional videotape. For immunohistology, embryos were either heat-fixed or fixed using modified Stefanini's fixative and stained with antibodies essentially as described elsewhere [66]. In situ hybridization with digoxygenin-labeled antisense RNA was performed as described before [67]. Nile Red staining was performed after Welte et al. [33]. The following antibodies were used: rabbit anti-Slam [28]; mouse anti-Neurotactin (Developmental Studies Hybridoma Bank, University of Iowa, Iowa City, Iowa, United States), mouse anti-Arm [41], rabbit anti-Arm^central^ (Keßler and Müller, unpublished data), and mouse anti-KlarM [36]. All secondary fluorochrome-conjugated antibodies were from Jackson ImmunoResearch (West Grove, Pennsylvania, United States) or Molecular Probes (Eugene, Oregon, United State). Imaging was performed on a Leica TCS Confocal microscope, and image processing was performed with Adobe Photoshop (Adobe Systems, San Jose, California, United States).

### Molecular biology.

Molecular cloning was performed following standard protocols. RT-PCR was performed using OneStep RT PCR kit (Qiagen, Valencia, California, United States) with embryonic poly-A^+^ RNA as template. Primer sequences are available upon request. Products from RT-PCR were directly sequenced (Seqlab, Göttingen, Germany). Northern blotting was performed using poly-A^+^ RNA isolated from 0- to 4-h-old embryos obtained from *w^1118^* or *ago2^dop1^* homozygotes. The *ago2* rescue construct was generated using the full-length *ago2* cDNA [9], which was cloned into CaTub_MatpolyA (a gift of D. Ferrandon, Strasburg, France). For DNA sequencing, fragments of *ago2* genomic DNA were amplified using Pfu-polymerase (Promega, Madison, Wisconsin, United States) and cloned using the TOPO-cloning kit (Invitrogen, Carlsbad, California, United States). DNA sequencing was performed by Seqlab, and sequences were examined using Lasergene (DNASTAR) software (Madison, Wisconsin, United States).

### Immunoblotting and immunoprecipitation.

For preparation of protein extracts, 0- to 12-h-old embryos were dechorionated and homogenized in CHAPS lysis buffer (20 mM Tris [pH 8], 150 mM NaCl, 10% glycerol, 2 mM EDTA, 10 mM CHAPS) containing proteinase inhibitors (pepstatin, aprotenin, leupeptin, pefabloc, and lactacystin). The solution was kept on ice for 10 min and then centrifuged for 15 min at 4 °C at 13,000 rpm. The supernatant was transferred into a new reaction tube, and the protein concentration was measured using the Bradford assay (Bio-Rad, Hercules, California, United States). For SDS-PAGE electrophoresis and Western blotting, 30 μg of protein was boiled in SDS sample buffer and loaded onto a discontinuous, horizontal SDS-polyacrylamide gel. Ovaries were dissected in PBS and directly boiled in SDS-sample buffer. The separation of the proteins was performed in electrophoresis buffer [68]. The proteins were blotted on nitrocellulose membrane (Schleicher & Schuell, Keene, New Hampshire, United States) and detected using the following antibodies: rabbit anti-Ago1 (1:250, Abcam, Cambridge, United Kingdom), rabbit anti-Dcr-1 (1:500, Abcam), rabbit anti-Loqs (1:2,000; [42]), rabbit anti-Ago2^Cterm^ (1:5,000, generated in the Müller laboratory against the carboxyl-terminal peptide CIVPEFMKKNPMYFV), rabbit anti-Ago2 (1:300, gift of Q. Liu), and rabbit anti-GFP antibody (Molecular Probes). As secondary antibodies, goat anti-rabbit antibodies conjugated with HRP were used at 1:10,000 (Jackson) and detected using the BM Chemiluminescence Blotting Substrate (Roche, Basel, Switzerlan).

For immunoprecipitation, the rabbit anti-Ago2^Cterm^ was covalently bound to the coupling gel using AminoLink Plus Immobilization Kit (Pierce Biotechnology, Rockford, Illinois, United States). Extracts containing 500 μg of protein were used for each reaction. The extract was incubated with the covalently bound beads for 1.5 h at 4 °C. The beads were washed, boiled in SDS-sample buffer, and subjected to SDS-PAGE and Western blotting.

### Scanning electron microscopy.

The heads of 2- to 4-day-old adult female flies were fixed in 30% ethanol. The specimen were dehydrated with a graded ethanol and acetone series and gradually transferred into tetra-methyl-silane. After overnight incubation, the specimen were air-dried, mounted onto double-stick carbon tape, and sputtered. Scanning electron microscopy imaging was performed on a LEO scanning electron microscope, and images were processed with Adobe Photoshop.

### Computational analyses of genome data.

For *Drosophila* species, genomic sequences corresponding to the *ago2* locus were retrieved using the UCSC Genome Browser (http://genome.ucsc.edu) and analyzed for exon-intron boundaries with the MIT Genscan Web server (http://genes.mit.edu/GENSCAN.html). For each species, the predicted exon containing sequences corresponding to the conserved stretch of D. melanogaster exon 3 was analyzed in detail (Figure S3A). For *D. simulans,* the splice-site prediction was verified by RT-PCR analysis and sequencing (Y. Guo and M. A. Welte, unpublished data). For *D. mojavensis,* the Genscan prediction of the 5′ end of exon 3 was likely incorrect because it would exclude an aa stretch conserved in other Ago2 proteins. In this case the prediction shown in Figure S3 is the glutamine-rich open reading frame directly 5′ to the conserved stretch. For the yellow fever mosquito, Aedes aegypti, we found that the whole genome shotgun sequence AAGE01113413 contains the likely homolog of *ago2* exons 3 and 4. An open reading frame identified in this genomic sequence has the capacity to encode a glutamine-rich region directly 5′ to the exon 3 sequences (Figure S3B). For the African malaria mosquito, Anopheles gambiae, the sequences corresponding to exons 3 and 4 of *D. melanogaster ago2* were identified in the genomic sequence (using the UCSC Genome Browser). This region of the genome is apparently expressed as it is also represented in several spliced EST sequences, including BM597722. These ESTs were used to determine the 5′ extent of the sequence shown in Figure S3B. For the honeybee, *Apis mellifera,* the NCBI-predicted protein XP_395048.2 was used.

## Supporting Information

Figure S1 *Klar* Is Still Functional in *ago2^dop1^* Embryos Embryos from wild-type (A), *klar^B^* (B), and *ago2^dop1^* (C) mothers were injected with the transcription inhibitor alpha-amanitin to prevent expression of Halo. In early cycle 14, this global inhibition of transcription causes a droplet transport defect very similar to deletion of *halo* [35]. In *klar^B^* embryos, Klar function is absent, and the peripheral cytoplasm becomes transparent because lipid droplets accumulate basally. In both wild-type and *ago2^dop1^* embryos, droplets accumulate apically resulting in an opaque periphery.
(2.0 MB TIF)

Figure S2 Expression of *halo* Transcripts in Wild-Type and *ago2^dop1^* Embryos Full-length *halo* digoxygenin-labeled antisense in situ probe was used for in situ hybridization of control *(w^1118^)* embryos (A, C, E, G) and embryos from *ago2^dop1^* homozygous mothers (B, D, F, H). (A, B) Syncytial blastoderm; (C, D) early cycle 14; (E, F) mid-cellularization stages; and (G, H) late cellularization (fast phase). Note that *halo* exhibits strictly zygotic expression, which is downregulated at the end of cellularization [35]. This expression pattern is largely unimpaired in *ago2^dop1^* mutants.
(1.8 MB TIF)

Figure S3 Cellularization in *ago2^51B^* and *ago2^414^* Mutant Embryos Is Unimpaired Embryos were obtained from Oregon R (wild-type) (A–C), or *ago2^dop1^* (D–F), *ago2^51B^* (G–I), and *ago2^414^* (J–L) homozygous mothers, fixed, and immunolabeled for Arm (red), Nrt (green), and DNA (blue). Consecutive time points during cellularization are shown from left to right panels for each genotype. Note that cellularization occurs normal in *ago2^51B^* and *ago2^414^* mutant embryos. The kinetics of membrane formation in *ago2^51B^* and *ago2^414^* mutant embryos is very similar to that in the wild-type (unpublished data).
(6.3 MB TIF)

Figure S4 Distribution of Lipid Droplets in *ago2^dop1^* and *ago2^51B^* Mutant Embryos Was Analyzed by Nile Red Staining Embryos at the extended germband stage were fixed and stained with the lipid droplet specific dye Nile Red.
(A) In the wild-type, Nile Red staining is uniformly distributed.
(B) In *ago2^dop1^* mutant embryos, the outer cell layers are devoid of staining indicative of failure of lipid droplets to move apically (compare to Figure 2).
(C) Embryos from *klar* mutant mothers displays a similar failure of apical transport.
(D) *ago2^51B^* mutant embryo displays a wild-type distribution of lipid droplets, indicated by uniform Nile Red staining.
(6.1 MB TIF)

Figure S5 Amino-Terminal GRRs in Ago2 Proteins from Different Insects The protein sequence encoded in *ago2* exon 3 of D. melanogaster is listed at the top. The corresponding region of Ago2 predicted from genomic sequences are shown for six additional *Drosophila* species, the malaria mosquito A. gambiae, the yellow fever mosquito A. aegytpi, and the honeybee A. mellifera. In each case, a bipartite structure is apparent: an amino-terminal glutamine-rich region (glutamines indicated in red and bold) of variable sequence followed by a conserved stretch at the 3′ end of exon 3 (purple). In many instances, the glutamine-rich regions contain multiple imperfect copies of distinct repeats (yellow, blue, or green). The 5′ extent of the region to be included was based on EST evidence *(A. gambiae),* an existing prediction by NCBI using GNOMON *(A. mellifera),* or splice-site predictions using Genscan (*Drosophila* species except D. mojavensis). Indented sequence is the portion of the protein employed to compute glutamine content in Table 3.
(77 KB DOC)

Figure S6 The *ago2^dop1^* Mutation Does Not Interfere with the Activity of the miRNA *bantam (ban)*
To test for miRNA activity, we employed an eye-based reporter assay for the function of *ban*. *ban* negatively regulates the expression of the proapoptotic regulator Hid [69]. Expression of *ban* in the eye using *GMR::Gal4* does not grossly affect eye development (A). Expression of *GMR::hid* induces cell death in the retina and thus results in a strongly reduced eye size (B). This phenotype is only slightly suppressed by the EP insertion *ban^EP3266^* (banEP) alone (C) but strongly suppressed by overexpression of *ban^EP3266^* using *GMR::Gal4* (D). To test interference of *ago2^dop1^* with *ban* activity, we performed the same experiment in *ago2^dop1^* heterozygous (E), *ago2^dop1^* homozygous (F) or *ago2^dop1^* hemizygous (G) genetic backgrounds. In neither case did we detect a suppression of *ban* activity, which should result in a reversion to the *GMR::hid* phenotype and produce a strong reduction of the size of the eye. The increased activity of *ban* in *ago2^dop1^* homozygous or hemizygous flies is explained by two copies of the *ban^EP3266^* insertion present in these animals. We conclude that *ago2^dop1^* does not inhibit the activity of *ban* in regulating Hid expression in this assay. Interestingly, in an *ago2^51B^* homozygous background (H), activity of *ban* seems to be slightly reduced: in the presence of two copies of *ban^EP3266^* the size of the eye is considerably smaller as compared to *ago2^51B^* heterozygotes (I), which contain only one copy of *ban^EP3266^*. This result suggests that Ago2 might be involved in the activity of *ban* to downregulate Hid. The genotypes are indicated above each panel, respectively. *TM6* and *MKRS* correspond to balancer chromosomes.
(2.0 MB TIF)

Figure S7 Densitometric Analysis of Independent Western Analyses Measuring Ago1, Dcr-1, and Loqc Levels in Protein Extracts of Mutant and Wild-Type Cells The quantification of the Western blots was performed from scanned images in a linear range using ImageJ from NIH Image. The columns represent the integrated density levels for independent Western blots detecting Ago1 (*n* = 3), Dcr-1 (*n* = 2), and Loqs (*n* = 3) in wild-type and *ago2* mutants (error bars show standard deviation). All values have been normalized against the α-tubulin loading control.
(168 KB DOC)

### Accession Numbers

GenBank (http://www.ncbi.nlm.nih.gov/Genbank) accession numbers are whole genome shotgun sequence AAGE01113413 (59568258), BM597722 (18895825), and protein XP_395048.2 (66517254).

The National Center for Biotechnology Information (NCBI) (http://www.ncbi.nlm.nih.gov) accession numbers are Argonaute 2 (39683), Argonaute 1 (36544), Dicer-1 (42693), Dicer-2 (36993), Loquacious (34751), Klarsicht (38067), Halo (33334), Wingless (34009), Engrailed (36240), Slam (33890), Armadillo (31151), and DIAP1 (39753).

## Abbreviations

aa: amino acid(s)
dsRNA: double-stranded RNA
GRR: glutamine-rich repeat
MBT: midblastula transition
miRNA: microRNA
RISC: RNA-induced silencing complex
RNAi: RNA interference
siRNA: small interfering RNA
