# Supplementary figures and images for: Overlapping Functions of Argonaute Proteins in Patterning and Morphogenesis of Drosophila Embryos
Source: PLoS Genet. 2006 Aug 25;2(8):e134. doi: 10.1371/journal.pgen.0020134 (PMC1557783; doi:10.1371/journal.pgen.0020134)

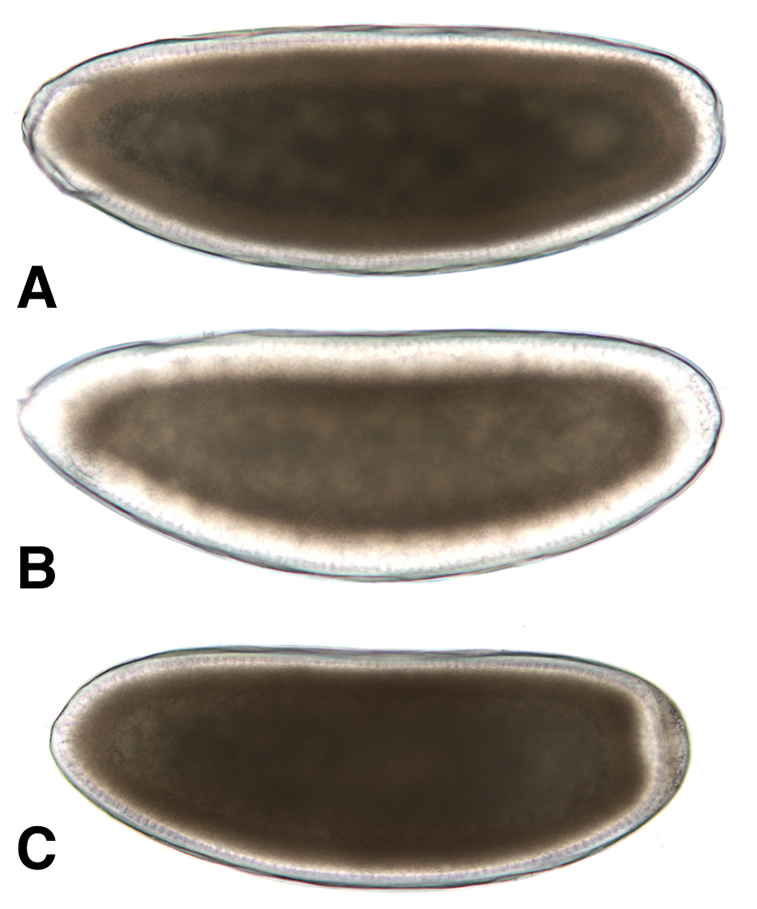

Supplement: Figure S1 — Embryos from wild-type (A), klarB (B), and ago2dop1 (C) mothers were injected with the transcription inhibitor alpha-amanitin to prevent expression of Halo. In early cycle 14, this global inhibition of transcription causes a droplet transport defect very similar to deletion of halo [35]. In klarB embryos, Klar function is absent, and the peripheral cytoplasm becomes transparent because lipid droplets accumulate basally. In both wild-type and ago2dop1 embryos, droplets accumulate apically resulting in an opaque periphery. (2.0 MB TIF) [file pgen.0020134.sg001.tif]

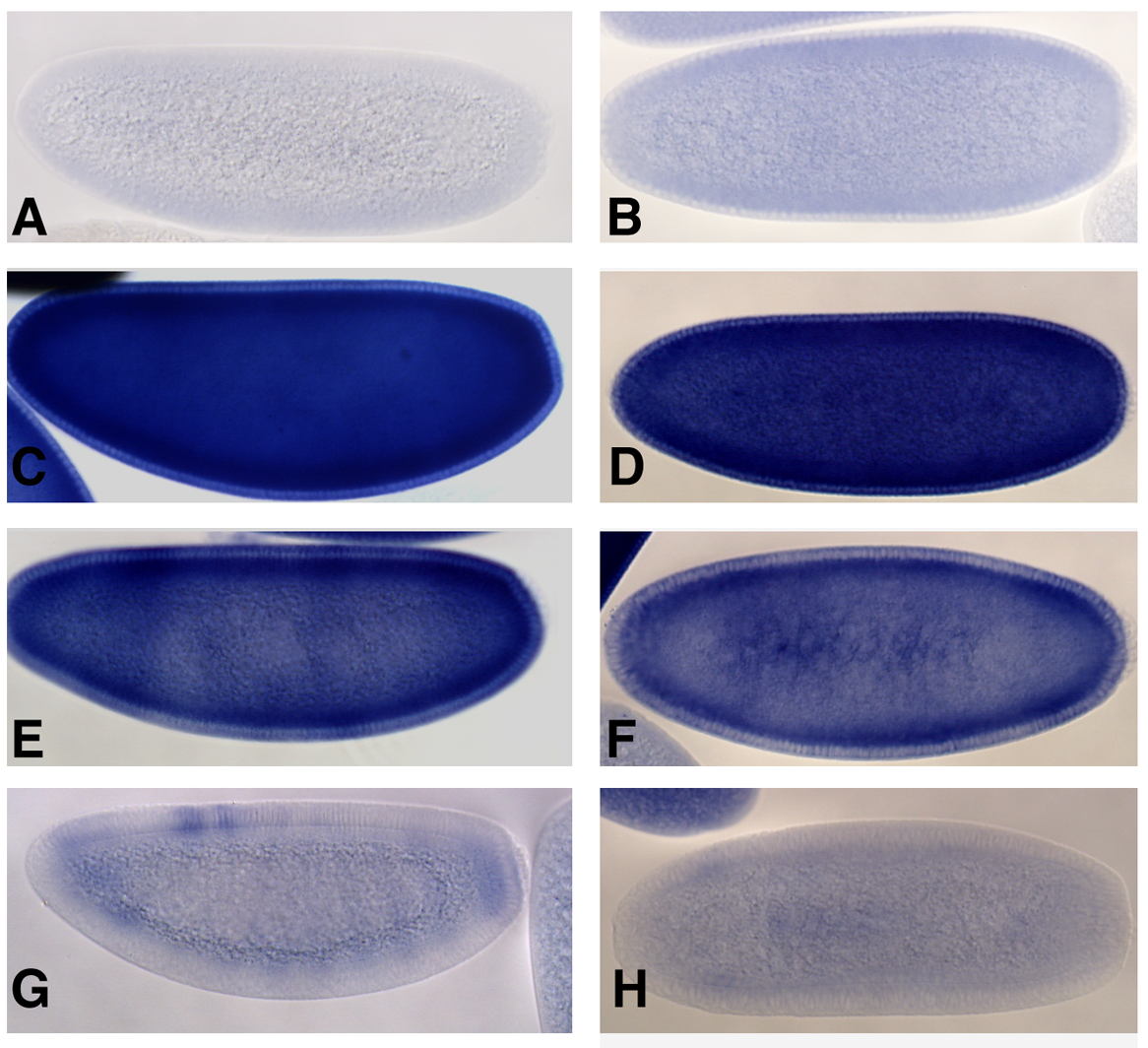

Supplement: Figure S2 — Full-length halo digoxygenin-labeled antisense in situ probe was used for in situ hybridization of control (w1118) embryos (A, C, E, G) and embryos from ago2dop1 homozygous mothers (B, D, F, H). (A, B) Syncytial blastoderm; (C, D) early cycle 14; (E, F) mid-cellularization stages; and (G, H) late cellularization (fast phase). Note that halo exhibits strictly zygotic expression, which is downregulated at the end of cellularization [35]. This expression pattern is largely unimpaired in ago2dop1 mutants. (1.8 MB TIF) [file pgen.0020134.sg002.tif]

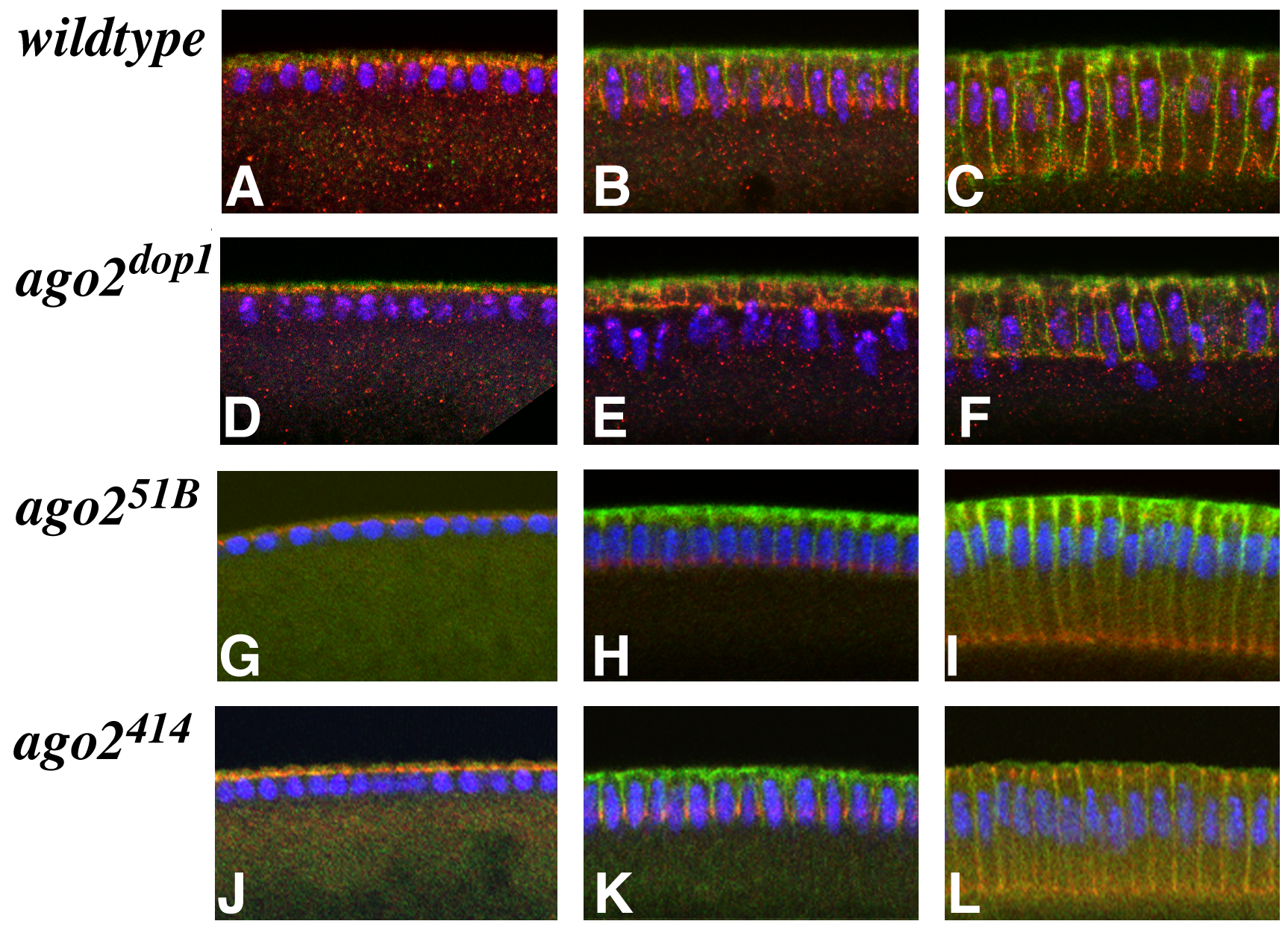

Supplement: Figure S3 — Embryos were obtained from Oregon R (wild-type) (A–C), or ago2dop1 (D–F), ago251B (G–I), and ago2414 (J–L) homozygous mothers, fixed, and immunolabeled for Arm (red), Nrt (green), and DNA (blue). Consecutive time points during cellularization are shown from left to right panels for each genotype. Note that cellularization occurs normal in ago251B and ago2414 mutant embryos. The kinetics of membrane formation in ago251B and ago2414 mutant embryos is very similar to that in the wild-type (unpublished data). (6.3 MB TIF) [file pgen.0020134.sg003.tif]

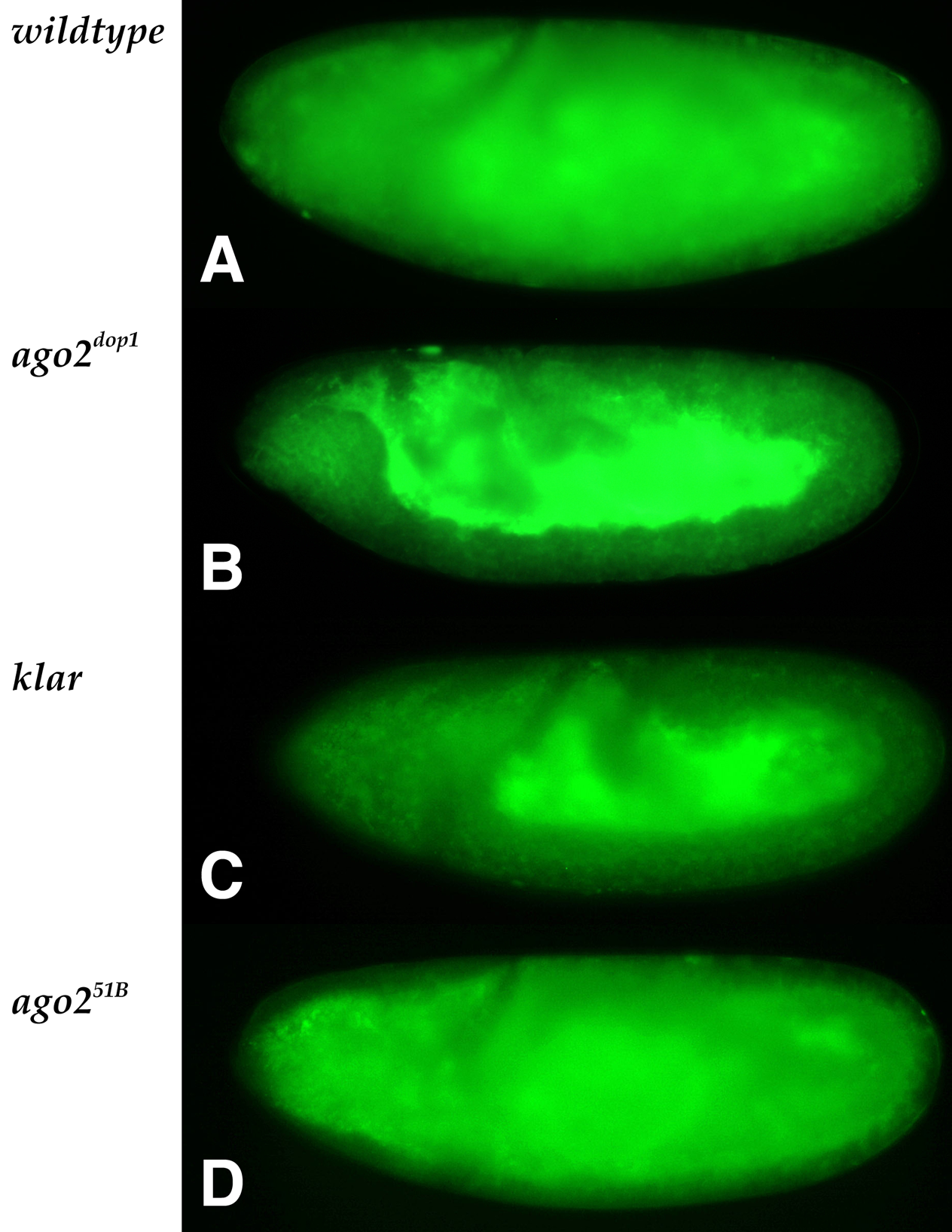

Supplement: Figure S4 — Embryos at the extended germband stage were fixed and stained with the lipid droplet specific dye Nile Red. (A) In the wild-type, Nile Red staining is uniformly distributed. (B) In ago2dop1 mutant embryos, the outer cell layers are devoid of staining indicative of failure of lipid droplets to move apically (compare to Figure 2). (C) Embryos from klar mutant mothers displays a similar failure of apical transport. (D) ago251B mutant embryo displays a wild-type distribution of lipid droplets, indicated by uniform Nile Red staining. (6.1 MB TIF) [file pgen.0020134.sg004.tif]

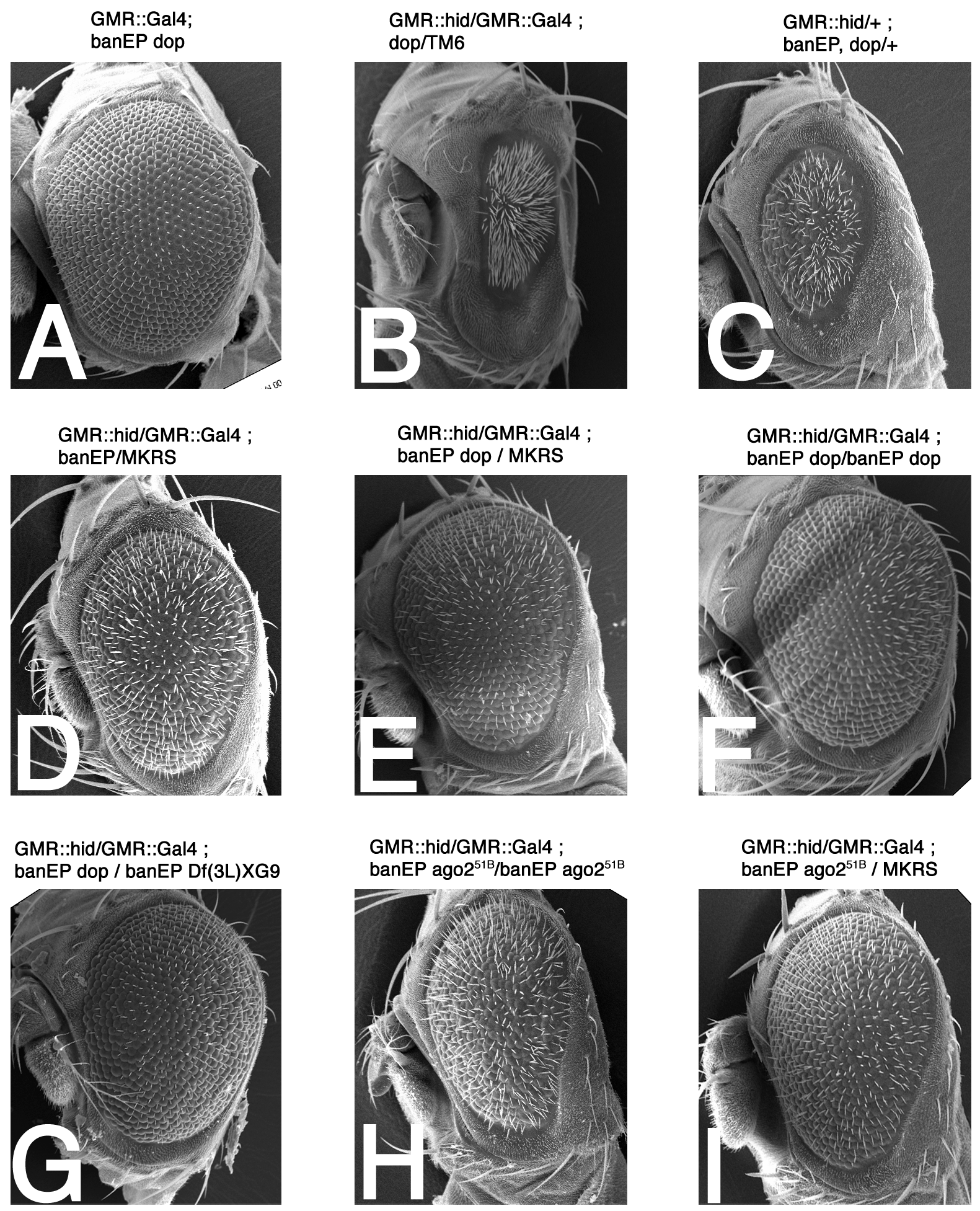

Supplement: Figure S6 — To test for miRNA activity, we employed an eye-based reporter assay for the function of ban. ban negatively regulates the expression of the proapoptotic regulator Hid [69]. Expression of ban in the eye using GMR::Gal4 does not grossly affect eye development (A). Expression of GMR::hid induces cell death in the retina and thus results in a strongly reduced eye size (B). This phenotype is only slightly suppressed by the EP insertion banEP3266 (banEP) alone (C) but strongly suppressed by overexpression of banEP3266 using GMR::Gal4 (D). To test interference of ago2dop1 with ban activity, we performed the same experiment in ago2dop1 heterozygous (E), ago2dop1 homozygous (F) or ago2dop1 hemizygous (G) genetic backgrounds. In neither case did we detect a suppression of ban activity, which should result in a reversion to the GMR::hid phenotype and produce a strong reduction of the size of the eye. The increased activity of ban in ago2dop1 homozygous or hemizygous flies is explained by two copies of the banEP3266 insertion present in these animals. We conclude that ago2dop1 does not inhibit the activity of ban in regulating Hid expression in this assay. Interestingly, in an ago251B homozygous background (H), activity of ban seems to be slightly reduced: in the presence of two copies of banEP3266 the size of the eye is considerably smaller as compared to ago251B heterozygotes (I), which contain only one copy of banEP3266. This result suggests that Ago2 might be involved in the activity of ban to downregulate Hid. The genotypes are indicated above each panel, respectively. TM6 and MKRS correspond to balancer chromosomes. (2.0 MB TIF) [file pgen.0020134.sg006.tif]
