# Supplementary material for: Overlapping Functions of Argonaute Proteins in Patterning and Morphogenesis of Drosophila Embryos
Source: PLoS Genet. 2006 Aug 25;2(8):e134. doi: 10.1371/journal.pgen.0020134 (PMC1557783; doi:10.1371/journal.pgen.0020134)
Supplement: Figure S5 — The protein sequence encoded in ago2 exon 3 of D. melanogaster is listed at the top. The corresponding region of Ago2 predicted from genomic sequences are shown for six additional Drosophila species, the malaria mosquito A. gambiae, the yellow fever mosquito A. aegytpi, and the honeybee A. mellifera. In each case, a bipartite structure is apparent: an amino-terminal glutamine-rich region (glutamines indicated in red and bold) of variable sequence followed by a conserved stretch at the 3′ end of exon 3 (purple). In many instances, the glutamine-rich regions contain multiple imperfect copies of distinct repeats (yellow, blue, or green). The 5′ extent of the region to be included was based on EST evidence (A. gambiae), an existing prediction by NCBI using GNOMON (A. mellifera), or splice-site predictions using Genscan (Drosophila species except D. mojavensis). Indented sequence is the portion of the protein employed to compute glutamine content in Table 3. (77 KB DOC) [file pgen.0020134.sg005.doc]

### Figure S5A: Drosophila species

### *D. melanogaster*

SR**QQ**PSTSSGGSRASGF

**QQ**GG**QQQ**KS**Q**DAEGWTA**Q**KK**Q**GK**QQ**V**Q**GWTK**Q**G**Q**

**Q**GGH**QQ**GR**Q**G**Q**DGGY**QQ**RPPG**QQ**

**Q**GGH**QQ**GR**Q**G**Q**EGGY**QQ**RPPG**QQ**

**Q**GGH**QQ**GR**Q**G**Q**EGGY**QQ**RPSG**QQ**

**Q**GGH**QQ**GR**Q**G**Q**EGGY**QQ**RPPG**QQ**

**Q**GGH**QQ**GR**Q**G**Q**EGGY**QQ**RPSG**QQ**

**Q**GGH**QQ**GR**Q**G**Q**EGGY**QQ**RPPG**QQ**

**Q**GGH**QQ**GR**Q**G**Q**EGGY**QQ**RPPG**QQ**

**Q**GGHE**Q**GR**Q**G**Q**EGGY**QQ**RPSG**QQ**

**Q**GGH**QQ**GR**Q**G**Q**EGGY**QQ**RPSG**QQ**

**Q**GGH**QQ**GR**Q**G**Q**EGGY**QQ**RPSG**QQ**

**Q**GGH**QQ**GR**Q**G**Q**EGGY**QQ**RPPG**QQ**PN**Q**T**Q**S**Q**G**Q**Y**Q**SRGPP**QQQQ**AAPLPLPP**Q**

PAGSIKRGTIGKPG**Q**VGINYLDLDLSKMPSVAYHYDVKIMPERPKKFYR**Q**AFE**Q**FRVD**Q**LGGAVLAYDGKASCYSVDKLPLNS**Q**NPEVT

### *D. simulans*

GLE

**QQ**PSTSSGGG**Q**K**Q**KF**Q**GWTG**Q**KT**Q**G**Q**ARDGSGY**QQQ**G**Q**WRPA**Q**G**QQ**RG**QQQ**G**Q**EGGY**QQ**RPPA**QQQ**GGH**QQ**GP**Q**GWPA**Q**G**QK**

GGY**QQ**GG**Q**R**Q**Y

GGY**QQ**G**Q**GGY**Q**T**Q**S**Q**G**Q**Y**Q**SRGPP**Q**

**QQ**PSTSSGGG**Q**K**Q**KF**Q**GWAG**Q**KT**Q**G**Q**ARDGSVD**QQQ**G**Q**WRPA**Q**GP**Q**RG**QQQ**G**Q**EGGY**QQ**RPPA**QQQ**GGH**QQ**GP**Q**GRPA**Q**G**Q**K

GGY**QQ**G**Q**GGY**Q**T**Q**S**Q**G**Q**Y**Q**SRGPP**Q**

**QQQ**AAPLPLPP**Q**

PEGSIKRGTIGRPG**Q**VAINYLDLDMSKMPSVAYHYDVKIMPERPTKFYR**Q**AFE**Q**FRMD**Q**LGGAILAFDGKASCYSVDKLPLNT**Q**NPEVT

### *D. yakuba*

LG**QQ**AAR**QQ**PTSSGW**Q**G**Q**G**Q**R**Q**GA**Q**

G**Q**G**QQ**GGF**QQ**RPPA**QQ**

G**Q**G**QQ**GGY**QQ**RPPAN**Q**

G**Q**G**QQ**GGS**QQ**RPPA**QQ**

G**Q**G**QQ**GGY**QQ**RPPAN**Q**

G**Q**G**QQ**GGY**QQ**RPPG**QQ**

G**Q**G**QQ**GGF**QQ**RPPA**QQ**

G**Q**G**QQ**GGY**QQ**RPPAN**Q**

G**Q**G**QQ**GGY**QQ**RPPA**QQ**

G**Q**G**QQ**GGY**QQ**RPPAN**Q**

G**Q**G**QQ**GGY**QQ**RPPG**QQ**

G**Q**G**QQ**GGF**QQ**RPPA**QQ**

G**Q**G**QQ**GGY**QQ**RPSAN**Q**

G**Q**G**QQ**GGY**QQ**RPPA**QQ**

G**Q**G**QQ**GGY**QQ**RPPA**QQ**

G**Q**G**QQ**GGY**QQ**RPPG**QQ**

G**Q**G**QQ**GGY**QQ**RPPA**QQ**HGA**Q**S**Q**G**Q**Y**Q**SRGPP**QQQQ**AVALPP**Q**

PAGSIKRGTIGKPG**Q**VGVNYLDIDMSKMPPVAYHYDVRIMPERPKKFYRHAFE**Q**FRMN**Q**LGGAIVAFDGRASCYSVDKLPVKS**Q**NPEVT

### *D. ananassae*

SSGAPVG

**Q**P**Q**R**QQ**GGGG**Q**GA**QQ**RNPL**QQ**LEP**Q**VGPTPS**Q**G**QQ**G**Q**GGWSRVPS**QQ**PGRS

**QQQQ**SHG**Q**GG**QQ**

**QQQQ**SHGKGG**QQ**

R**Q**PY**Q**G**Q**GG**QQ**

R**Q**PY**Q**G**Q**GG**QQ**

R**Q**PY**Q**G**Q**GG**QQ**

RY**QQ**GSSGF**Q**GGN

**QQ**RP**QQ**G**Q**PGT

**QQ**RP**QQ**G-PGP

S**Q**GGYR**QQ**GSHP**Q**P**Q**RS**Q**

GGAVAPSL

PAGSIKRGTIGRPGEVAVNYLDINMEKMPATAYHYDVKIMPERPKKFYR**Q**AFE**Q**YRVN**Q**LGGAIAAYDGKASCYSVDKLKTNS**Q**NPEVT

### *D. pseudoobscura*

AAAGAPGA**Q**NRPT**Q**PARPPATPTPSTSSAASGS

**Q**L**QQ**GGWRT**Q**DSH**QQ**RS**Q**AG**Q**GWGG**QQ**KGPR**Q**P

GGN**QQ**RGP**Q**K**Q**

GSG**Q**ERRP**QQQ**

GGG**QQ**RGP**QQQ**

GGG**QQ**RGPP**Q**K

**Q**GGAALPPL

PAGTMKRGTLGKPG**Q**VSVNYLDVNLDKMPAVAYHYDVKITPERPKKFYR**Q**AFE**Q**YRVEHLGGAIAAFDGRASAYSAVKLKCSS**Q**GHEV

### *D. virilis*

AAPAPSTSAAARENTSRL**Q**AGDDGVPAT

**Q**RP**QQ**HPES**Q**NP**Q**GEWRRP**Q**GG**QQ**RGP**QQQQQQ**YRP**QQQ**GG**QQ**R**Q**LGWK**Q**GE**Q**PRN**QQ**EESSG

**QQ**GGY**QQ**RGPSSGP**Q**GGA**Q**GGG

**QQ**GGY**QQ**RGPSSGP**Q**GGA**Q**GGG

**QQ**GGG**QQ**GGY**QQ**RGPSSGP**Q**GGA**Q**GGG

**QQ**GGY**QQ**RGLSSGP**Q**GGT**Q**DGG

**QQ**GGY**QQ**RGPSSGP**Q**GGA**Q**GGG

**QQ**GGY**QQ**RGPSSGP**Q**GGA**Q**GGG

**QQ**GGY**QQ**RGPSSGP**Q**GGA**Q**GGG

**QQ**GGG**QQ**GGY**QQ**RGPSSGP**Q**GGA**Q**GGG

**QQ**GGY**QQ**RGPPG**Q**

**QQ**GGY**QQ**RGP**Q**GP

**QQ**GGY**QQ**RGPPPA

**QQ**GGY**QQ**RGPP**Q**G

**QQ**GGY**QQ**RGPP**Q**G**Q**RT**Q**

GGALAPLLP

**Q**TGNIKRGTIGNAGRVGVNYLDVDISKMPDIAYHYDVTIVPERPKKFYRKAFEVFRAKHLDNGIAAFDGRKSCYSVDKLPNVTG**Q**VEV

### *D. mojavensis*

…**Q**AGDTRGPGP**Q**RSN**Q**PA**Q**GSGH**Q**RPASA

**Q**PS**Q**YRP**QQ**AG**QQ**

E**Q**GWSRAGPSGA**QQQ**GGN**Q**SGPPRRP**Q**GG**QQ**

E**Q**GWSRTGPSGA**QQQ**GGN**Q**SGPPKRP**Q**GG**QQ**

E**Q**GWSRAGPSGA**QQQ**GGD**Q**SGPPKRP**Q**GG**QQ**

E**Q**GWSRAGPSGA**QQQ**GGN**Q**SGPPKRP**Q**GG**QQ**

GGP**Q**SGY**QQ**RGPPGG**QQQ**RGPPGR**Q**GGYD**Q**RS**Q**

AGAMGPPLP

**Q**TGNIKRGTLGRAGFVDINYLDVDISKMPDIAYHYDVSIVPERPKKFYRNAFEEFRTKHLNNAIAAFDGRKSCYSVDKL**Q**NTTGEV

### Figure S5B: Other insects

### *Anopheles gambiae*

GAIG**Q**TVPGA

**QQQQQ**PP**QQQQQ**P**Q**P**QQQQQ**P**QQ**RP**QQQQQ**P**Q**P**QQ**RP**QQQQQQQ**P**Q**HP**QQQQQQQQQQ**KRP**QQQQQQQ**FPP**QQQQQQ**GNP**Q**KK**Q**KK**Q**

P**Q**SGGP**QQ**H**QQ**GPS**QQQQQ**HA**Q**K**Q**HEPP**QQQQQQ**P**Q**RP**QQQQQQQQQ**LP**QQQQQQQQQQQ**RP**QQQQQQQ**GPP**QQQQQQQQ**RPR**QQQ**

HKPAVPAAATGSAVHRDASVASTKSSSSGDGTL**Q**TIEENLGAMRIAKEKIRRTDLRPVL

VRRGAHGKRGEPVSVEANFFRLLLDKLKGTAYHYDVAIEPERPKKFYRPVFA**Q**FCRENYPGAMLAFDG**Q**KNAYTTRKLSDKKAKVV

### *Aedes aegypti*

NYVYLLI**Q**RENRRRVIDHCMILNARYLYIPCR

**Q**K**QQQQQQQ**P**QQQQQ**H**QQ**K**QQ**S**QQQQQQQQQQQ**RSKE**Q**GS**QQQQ**RP**QQQ**A**QQQQ**PS**QQQQQ**S

**Q**K**QQ**HP**QQQQQQ**RP**Q**KE**QQQ**F**QQ**D**Q**RP**QQQQQQ**F**Q**K**QQ**RP**QQQ**E**QQQQ**RP**QQQ**G**Q**SWRP**Q**

SHDPSPASGSHSHSSSPSHAAALERVEEDFSKIKIDK**Q**KIHSSALLPVLMRPNAH

GTRGRAIKVEVNYI**Q**LLLERLIPTAYHYDVDI**Q**PAASRKW**Q**RLAFSEFTK**Q**MFPNHGFAFDGHKNAYAARRL**Q**ADVYE**Q**EVKV

### *Apis mellifera*

kkk**q**nv**q**nhphssis

**qqq**snpd**qqq**npsgs**qq**vhs**qqqq**sdis**q**k

hacpk**qq**sdsp**qqq**g**q**tyd**qqqq**h-sss**qq**

shs**q**k**qq**snsp**qqq**awkln**qqqqq**-ssp**qq**

phs**q**r**qq**ldsp**qqq**awrln**qqqqq**-ssp**qq**

phs**q**r**qq**snsp**qqq**vrrpn**qqqq**y-ssp**qq**

shs**q**r**qq**snsp**qqq**awkln**qqqqq**-ssp**qq**

phs**q**r**qq**snsp**qqq**awrln**qqqqq**-ssp**qq**

phs**q**r**qq**lnsp**qqq**awrln**qqqqqq**ssp**qq**

phs**q**r**qq**snsp**qqq**vrrpn**qqqq**y-ssp**qq**

shs**q**r**qq**snsp**qqq**awkpn**qqqqq**-ssp**qq**

phc**q**r**qq**snsp**qqq**vwrpn**qqqqq**hnsp**qq**

vsish**qq**

imsds

yee**qqq**snkpkfllrtvfeefrkk**q**cpkrypafdgrknaysakllpfgdkskeeeinV

### Legend

Amino-terminal glutamine-rich repeats in Ago2 proteins from different insects. The protein sequence encoded in *ago2* exon 3 of *D. melanogaster* is listed at the top. The corresponding region of Ago2 predicted from genomic sequences are shown for six additional *Drosophila* species, the malaria mosquito *A. gambiae*, the yellow fever mosquito *A. aegytpi*, and the honeybee *A. mellifera*. In each case, a bi-partite structure is apparent: an amino-terminal glutamine-rich region (glutamines indicated in red and bold) of variable sequence followed by a conserved stretch at the 3’ end of exon 3 (purple). In many instances, the glutamine-rich regions contain multiple imperfect copies of distinct repeats (yellow, blue, or green). The 5’ extent of region to be included was based on EST evidence (*A. gambiae*), an existing prediction by NCBI using GNOMON (*A. mellifera*), or splice site predictions using Genscan (*Drosophila* species except *D. mojavensis*). Indented sequence is the portion of the protein employed to compute glutamine content in Table 3.
