# Supplementary material for: Overlapping Functions of Argonaute Proteins in Patterning and Morphogenesis of Drosophila Embryos
Source: PLoS Genet. 2006 Aug 25;2(8):e134. doi: 10.1371/journal.pgen.0020134 (PMC1557783; doi:10.1371/journal.pgen.0020134)
Supplement: Figure S7 — The quantification of the Western blots was performed from scanned images in a linear range using ImageJ from NIH Image. The columns represent the integrated density levels for independent Western blots detecting Ago1 (n = 3), Dcr-1 (n = 2), and Loqs (n = 3) in wild-type and ago2 mutants (error bars show standard deviation). All values have been normalized against the α-tubulin loading control. (168 KB DOC) [file pgen.0020134.sg007.doc]

Meyer et al. Figure S7
